# Transcription Factor *Amr1* Induces Melanin Biosynthesis and Suppresses Virulence in *Alternaria brassicicola*

**DOI:** 10.1371/journal.ppat.1002974

**Published:** 2012-10-25

**Authors:** Yangrae Cho, Akhil Srivastava, Robin A. Ohm, Christopher B. Lawrence, Koon-Hui Wang, Igor V. Grigoriev, Sharadchandra P. Marahatta

**Affiliations:** 1 Plant and Environmental Protection Sciences, University of Hawaii at Manoa, Honolulu, Hawaii, United States of America; 2 US DOE Joint Genome Institute, Walnut Creek, California, United States of America; 3 Virginia Bioinformatics Institute and Department of Biological Sciences, Virginia Tech, Blacksburg, Virginia, United States of America; Purdue University, United States of America

## Abstract

*Alternaria brassicicola* is a successful saprophyte and necrotrophic plant pathogen. Several *A. brassicicola* genes have been characterized as affecting pathogenesis of *Brassica* species. To study regulatory mechanisms of pathogenesis, we mined 421 genes *in silico* encoding putative transcription factors in a machine-annotated, draft genome sequence of *A. brassicicola*. In this study, targeted gene disruption mutants for 117 of the transcription factor genes were produced and screened. Three of these genes were associated with pathogenesis. Disruption mutants of one gene (*AbPacC*) were nonpathogenic and another gene (*AbVf8*) caused lesions less than half the diameter of wild-type lesions. Unexpectedly, mutants of the third gene, *Amr1*, caused lesions with a two-fold larger diameter than the wild type and complementation mutants. *Amr1* is a homolog of *Cmr1*, a transcription factor that regulates melanin biosynthesis in several fungi. We created gene deletion mutants of Δ*amr1* and characterized their phenotypes. The Δ*amr1* mutants used pectin as a carbon source more efficiently than the wild type, were melanin-deficient, and more sensitive to UV light and glucanase digestion. The AMR1 protein was localized in the nuclei of hyphae and in highly melanized conidia during the late stage of plant pathogenesis. RNA-seq analysis revealed that three genes in the melanin biosynthesis pathway, along with the deleted *Amr1* gene, were expressed at low levels in the mutants. In contrast, many hydrolytic enzyme-coding genes were expressed at higher levels in the mutants than in the wild type during pathogenesis. The results of this study suggested that a gene important for survival in nature negatively affected virulence, probably by a less efficient use of plant cell-wall materials. We speculate that the functions of the *Amr1* gene are important to the success of *A. brassicicola* as a competitive saprophyte and plant parasite.

## Author Summary

Induction of cell wall-degrading enzymes, secretion of toxins, enforcement of fungal cell wall architecture, and detoxification of host defense molecules are important or essential for the fungal infection of plants. Genes important for each of these functions have been identified in various fungal species. Understanding how these genes are coordinately regulated and how many regulators are involved in the process is a challenge. We recently discovered a transcription factor gene, *AbVf19*, which positively regulates hydrolytic enzyme-coding genes. Here, we report on another transcription factor gene, *Amr1*, which negatively regulates a subset of these genes during late-stage pathogenesis and positively regulates melanin biosynthesis during conidiogenesis. This study adds another dimension to the complex regulation and overall importance of hydrolytic enzyme genes during plant pathogenesis by necrotrophic fungi. In addition, this study provides an example on the evolutionary implication of virulence in the necrotrophic fungus, *A. brassicicola*. One transcription factor essential for long-term survival because of its role in melanin biosynthesis is used instead to suppress virulence. We speculate that the suppressive functions of *Amr1* contribute to the specialized adaptation of *A. brassicicola* as an efficient and successful facultative parasite.

## Introduction

*Alternaria brassicicola* is the causal agent of black spot disease of cultivated brassicas (e.g. cabbage, canola, mustard). Pathogenesis in necrotrophic fungi is generally described as a two-step process: killing host cells directly (necrosis) or inducing programmed cell death with toxins, then decomposing host tissues with cell wall-degrading enzymes. The importance of toxins in disease development by other necrotrophs has been clearly demonstrated [1], [2], [3], [4]. Several *A. alternata* pathotypes produce toxic, host-specific secondary metabolites that are essential for pathogenicity [5], [6], [7], [8]. Our knowledge of toxins produced by *A. brassicicola* is currently limited. In recent studies, Brassicicolin A emerged as the most selective phytotoxic metabolite produced in liquid cultures of *A. brassicicola* [9]. The genes responsible for biosynthesis of this toxin, however, have yet to be determined. Diterpenoid toxins called brassicenes have also been described from this fungus and linked to gene clusters in the *A. brassicicola* genome [10]. In addition, a cluster of five genes responsible for synthesis of the secondary metabolite depudecin, a histone deacetylation inhibitor was recently reported [11]. Mutation of the five genes abolished depudecin synthesis and caused only a small (10%) reduction in virulence compared to wild-type *A. brassicicola.* A weak protein toxin was also reported [12], [13] but the gene or genes responsible for its production have yet to be identified.

In addition to toxins, several *A. brassicicola* genes have been linked to pathogenesis [14], [15], [16], [17], [18], [19], [20]. These genes are involved in iron uptake, cell wall integrity, peroxisome-mediated redox homeostasis, hyphal fusion, hydrolytic enzymes, and signal transduction. All mutants of the pathogenesis-related genes showed either a reduction in virulence or loss of pathogenicity. For example, mutants of a mitogen-activated protein (MAP) kinase gene, *Amk1*, and its downstream transcription factor-coding gene, *AbSte12*, were nonpathogenic. The mutants of either gene also showed slow vegetative growth and impaired conidium maturation.

Bioinformatics analysis of the genome sequence of *A. brassicicola* identified many candidate genes important for pathogenesis. They included several genes that are essential for melanin synthesis. *Alternaria* species produce 1,8-dihydroxynaphthalene (1,8-DHN) melanin, like various other filamentous fungi [21], [22], [23]. It is heavily concentrated in the primary cell walls of conidia and in their septa [24]. Melanin is a ubiquitous pigment that plays an important role in protecting fungi from the damaging effects of environmental stress and so may be considered an advantageous adaptation. It increases the tolerance of fungi to UV irradiation [25], [26], [27], enzymatic lysis [28], and extreme temperatures [26], [29]. Melanin is also required for the mechanical penetration of host plants by other phytopathogenic fungi, such as *Magnaporthe grisea* and *Colletotrichum lagenarium* [30].

Synthesis of 1,8-DHN melanin requires polyketide synthase (*Pks1* in *Bipolaris oryzae* and *Pks18* in *Cochliobolus heterostrophus*), T4HN reductase (*Brn2*), scytalone dehydratase (*Scd1*), and T3HN reductase (*Brn1* or *Thr1*) genes identified in *B. oryzae* [31], *Col. lagenarium* [32], and *Coc.heterostrophus* [21], [33]. Expression of these genes is mainly regulated by the *Cmr1* transcription factor gene in *Col. lagenarium*. *Cmr1* homologs are regulators of the primary melanin biosynthesis pathway in several plant pathogenic fungi but virulence is not affected by the gene mutation in *M. grisea*, *Col. lagenarium*, or *Coc. heterostrophus* [21], [22].

For all pathogenesis-associated genes in *A. brassicicola* studied to date, their mutants have been either nonpathogenic, or less virulent than the wild type. In this study, we determined that mutants of the *Cmr1* homolog in *A. brassicicola* (*Amr1*) were melanin-deficient but more virulent than wild-type *A. brassicicola*. We defined virulence as quantitative host plant damage as measured by lesion diameter. The increase in virulence of the Δ*amr1* mutants suggested that the loss of the gene was beneficial to pathogenesis. We tested three research questions: whether melanin is important for pathogenesis, if mutants efficiently neutralize host defense chemicals, or if the mutants more efficiently utilize plant cell wall-associated materials such as pectin. Our study provides an example of a transcription factor gene that is important for survival in nature negatively regulates virulence in a necrotrophic plant pathogen. We speculate that the negative regulation of virulence by the *Amr1* gene contributed to *A. brassicicola*'s efficiency as a facultative plant pathogen while retaining characteristics of a robust saprophyte.

## Results

### Identification of putative transcription factor genes

The genome sequence of *A. brassicicola* has been determined by Washington University School of Medicine, Genome Sequencing Center, and is publicly available from DDBJ/EMBL/GenBank under accession ACIW00000000. In addition, the annotated genome is available in the context of other sequenced *Dothideomycetes* genomes through the interactive JGI fungal portal MycoCosm [34] at http://jgi.doe.gov/Abrassicicola. The *A. brassicicola* genome size is approximately 31.9 Mb, smaller than the average genome size of other dothideomycete genomes [35]. There are 10, 688 predicted genes in the assembled genome. We predicted 421 genes encoding putative transcription factors in the draft genome of *A. brassicicola* using Pfam searches [36]. These transcription factors excluded the core proteins necessary for the formation of transcription initiation complexes and RNA polymerases. We classified the transcription factors in 13 categories based on their functional domains (Table 1). Members of the largest group (221 of 421) contain at least one zinc-finger domain. The second largest group (72 of 421) contains fungal-specific transcription factor domains.

**Table 1:** Summary of *Alternaria brassicicola* transcription factor domains based on Pfam scans.

Description	gene	KO 1^a^	KO 2^b^	Pathogenesis-associated genes
Zn-finger domain	221	4	117	*AbVf8*, *AbVf19* c, *AbSte12* c, *AbPacC*, *AbPro1* c, *Amr1*
Fungal-specific TF	72	1	0	
phd finger	1	-	-	
Helix-turn-helix	29	1	-	
Regulatory receiver	23	5	-	
Leucine zipper	21	2	-	
High mobility box group	15	3	-	
Helix-loop-helix	13	5	-	
DNA binding myb	10	-	-	
Jumonji	7	-	-	
Homeobox	4	-	-	
B-Regulatory protein	3	-	-	
T-Regulatory protein	2	-	-	
Total	421	21	117	

a Genes of previously screened targeted deletion mutants [15].

b Genes of mutants created and screened in this study.

c Genes whose functions were previously characterized [15], [20].

### Discovery of pathogenesis-associated transcription factors

We previously created targeted deletion mutants for 22 transcription factor and signaling related-genes and reported the association of *AbSte12*, *AbPro1*, and *AbVf19* transcription factors with pathogenesis [15], [20]. In this present study, we generated targeted gene disruption mutants for an additional 117 transcription factor genes (Figure S1). All mutants corresponding to 109 of the 117 genes caused disease symptoms on host plants with lesion sizes similar to those caused by the wild type in multiple pathogenicity assays. Mutants of eight of 117 genes showed unusual changes in virulence. Among the eight mutants, five mutants also grew very slowly on nutrient rich medium. These mutants either failed to cause disease symptoms or their lesions did not expand beyond the initial infection site (Table S1). Gene disruption mutants of the *AbPacC* transcription factor (*abpacc*) were nonpathogenic (Table 2), unlike the severely reduced virulence in the loss-of-function mutants of its homologs in *Col. acutatum* [37] and *Sclerotinia sclerotiorum* [38] or no changes in *Ustilago maydis* [39]. Mutants corresponding to two other genes (*Amr1*and *AbVf8*) grew normally on nutrient rich media, were novel factors associated with pathogenesis, and are described further in this study.

**Table 2:** Loss of pathogenicity of the *abpacc* mutant and decreased virulence of Δ*abvf8* compared to wild-type *Alternaria brassicicola*.

		Lesion diameter (mm)			
Mutants	d.f.	wt	mutant	Decrease in lesion diameter	*p*-value
*abpacc-2*	8	11.6±3.7	0.0±0.0	-	1.39E-05
Δ*abvf8-2*	11	11.5±4.1	7.7±4.6	55.45%	1.05E-05
Δ*abvf8-3*	11	15.1±4.4	10.6±5.5	55.09%	2.00E-04
AbVf8-10*	11	15.8±4.2	15.8±3.9	0.00%	0.72

d.f. = degrees of freedom, wt = wild type, p-value = probability, AbVf8-10* is an ectopic mutant.

### The *Amr1* gene

In contrast to the loss-of-function mutants of other virulence genes studied thus far in this pathosystem, *Amr1* disruption mutants (*amr1*) caused larger lesions than wild-type *A. brassicicola* when green cabbage (*Brassica oleracea*) leaves were inoculated with the same number of conidia (data not shown, see below for detailed study). The predicted *Amr1* gene encodes a protein of 997 amino acids with two C_2_H_2_-zinc finger motifs and one fungal-specific Zn(II) binuclear motif. Of the 997 predicted amino acids, 869 (87%) were identical (*E*-value = 0) to the transcription factor *Cmr1* in the taxonomically closely related dothideomycete fungus, *Coc. heterostrophus* [21]. We named this *Cmr1* homolog in *A. brassicicola*, *Amr1* (Alternaria melanin regulation, GenBank accession number: JF487829). Only one copy of the gene was present in the draft genome sequence of *A. brassicicola* (http://jgi.doe.gov/Abrassicicola).

### Replacement mutants of the *Amr1* gene

**Figure 1:**
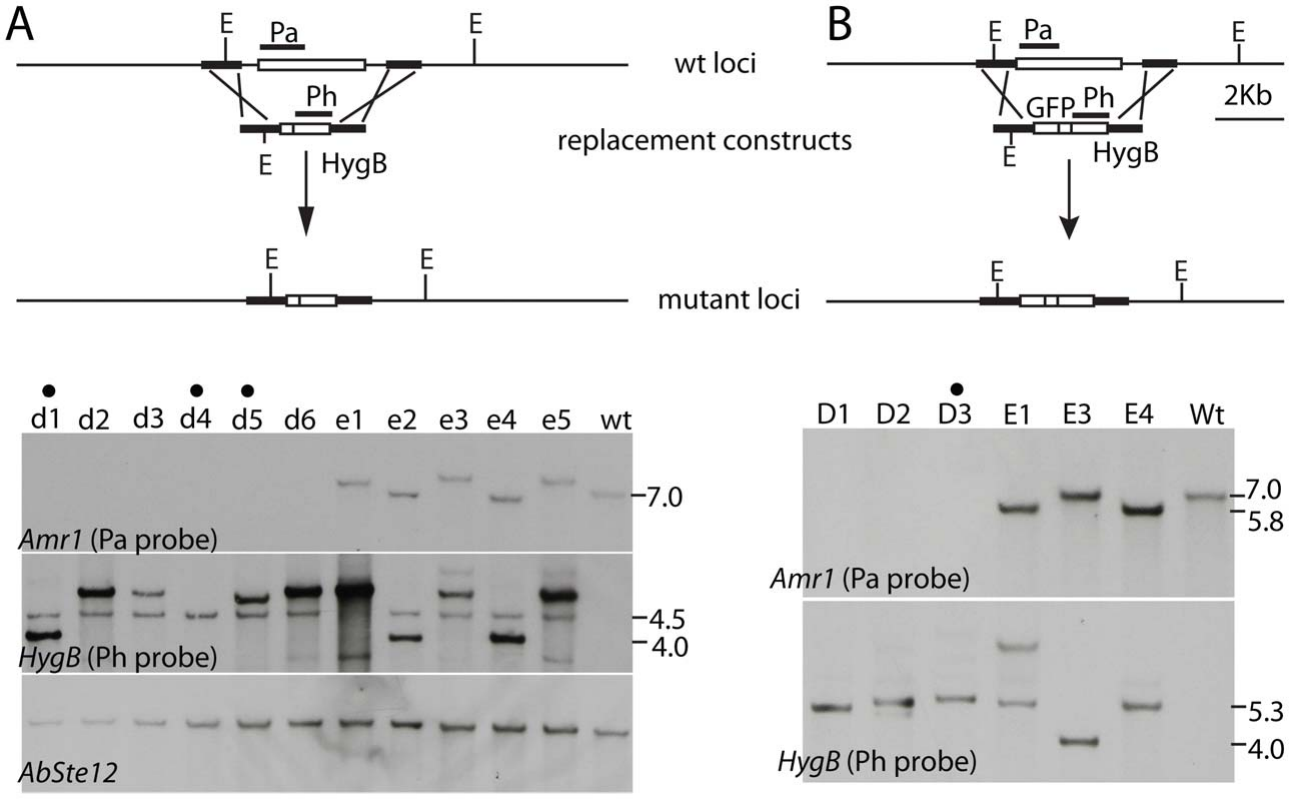
Verification of *Δamr1* deletion mutants. A. Replacement of the *Amr1* coding region with the selectable marker, Hygromycin B transferase (HygB) resistance cassette. On the HygB-probed blot, the expected 4.5 Kb band indicates a single copy insertion of the Hyg B resistance cassette in lane d4. Additional bands in the other lanes suggest multiple insertions of the cassette. B. Replacement of the *Amr1* coding region with a green fluorescent protein (GFP) coding region and a HygB cassette. The upper panel of both A and B is a schematic diagram of wild-type (wt) and mutant loci and the lower panel consists of five Southern blots showing loss of the *Amr1* gene in selected mutants. Dots (•) indicate lanes of mutants used in this study. Probe regions are marked by P_h_ and P_a_. Abbreviations: E = *Eco*RI enzyme digestion site. d1 = Δ*amr1-1*, d4* = *Δ*amr1-4*, d5* = * Δ*amr1-5*, and D3 = Δ*amr1:Amr1p-GFP.* e1-e5, E1, E3, and E4 are ectopic insertion mutants.

In order to confirm our results with gene disruption mutants and to perform additional studies, deletion mutants of *Amr1* (Δ*amr1*) were produced by replacing the coding region with either a Hygromycin B (HygB) resistance cassette, or a green fluorescent protein (GFP) coding sequence [40] plus a HygB resistance cassette. Southern hybridization confirmed that the *Amr1* gene was absent from nine melanin-deficient transformants but still present in the eight transformants producing melanin (Figure 1). We replaced the *Amr1* coding region with a single copy of the HygB resistance cassette in Δ*amr1-4* and three Δ*amr1:Amr1p-GFP* mutants (d4, D1, D2, and D3 in Figure 1). The replacement construct containing the GFP and HygB resistance cassette was designed so the *GFP* gene would be regulated by native promoter elements of the *Amr1* gene in the replacement mutants (Δ*amr1:Amr1p-GFP*) (Figure 1B). We complemented Δ*amr1-5* mutant with either the wild-type allele or a chimeric construct with the *TrpC* promoter to constitutively express the wild-type *Amr1* gene.

### Increased virulence of the Δ*amr1* mutants

**Figure 2:**
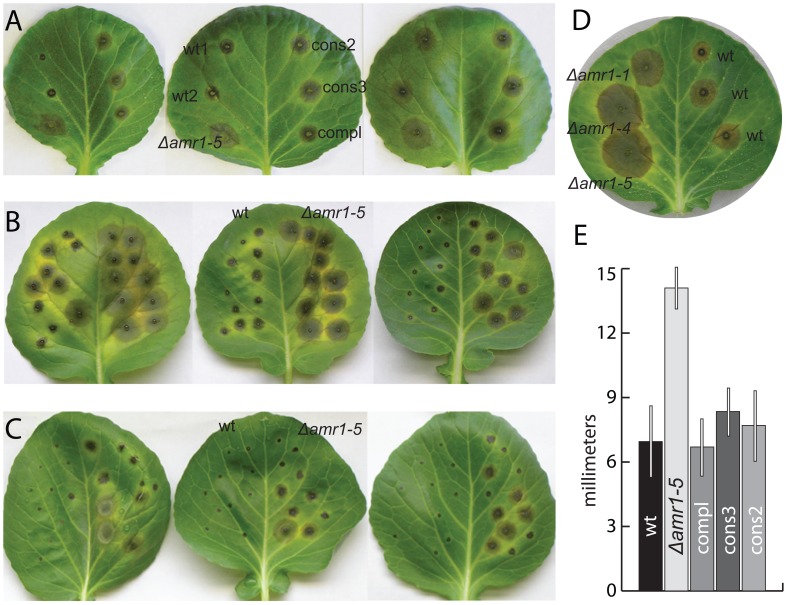
Increased virulence of Δ*amr1* mutants on individual host plants (*Brassica oleracea*). A. Lesions on *B. oleracea* 5 days after inoculation with ∼2,000 conidia of the wild type, a Δ*amr1* mutant, and three complemented mutants with two types of constructs. B. Lesions produced by ∼440 and ∼400 conidia respectively of the wild type (left) and a Δ*amr1* mutant (right). C. Lesions produced by ∼330 and ∼300 conidia respectively of the wild-type (left) and a Δ*amr1* mutant (right). The three images in panels A–C are replicate experiments with the same pattern of inoculation. Note that the size of lesions caused by the mutant is similar on all leaves, while lesions caused by the wild type are smaller on young leaves than on old leaves. Δ*amr1-1* and Δ*amr1-4* produced similar results. D. Pathogenicity assay results for three mutants (Δ*amr1-1*, Δ*amr1-4*, and Δ*amr1-5*) compared to the wild type. E. Pathogenicity assay results with average lesion diameter (mm) and standard deviation. Abbreviations: wt = wild-type *A. brassicicola*; Δ*amr1 = Amr1* deletion mutant; compl = mutant complemented with a native allele of the *Amr1* gene (Δ*amr1:Amr1*); cons = mutant complemented with chimeric constructs of the *TrpC* promoter and a native allele of the *Amr1* coding region (Δ*amr1:TrpCp-Amr1*).

Because the increase in virulence in *A. brassicicola* was unusual and unprecedented, we performed additional pathogenicity assays with four strains of gene deletion mutants (Δ*amr1-1*, Δ*amr1-4*, Δ*amr1-5*, and Δ*amr1:Amr1p-GFP*). All lesions caused by the mutants were 30–80% larger in diameter than the wild type in assays with 5- to-7-week-old plants (Table 3). Generally, wild-type *A. brassicicola* caused larger lesions on 5-week old plants than on 7-week-old plants and on older leaves compared to younger leaves. Interestingly, all Δ*amr1-*type mutants produced consistent lesion sizes regardless of plant age. Thus, the relative size of lesions caused by the mutants compared to the wild type was greater on 7-week-old plants than on 5-week-old plants and on younger leaves than on older leaves (Figure 2A and Table 3). The difference in lesion size was similar between assays on whole plants and assays on detached leaves, so for convenience we performed a subsequent assay on the detached leaves of 8-week-old plants. In this assay, we compared lesion sizes caused by a gene deletion mutant (Δ*amr1*), one strain from each of the two complemented mutants (Δ*amr1:Amr1* and Δ*amr1:TrpCp-Amr1*), and the wild type. Lesions caused by the deletion mutant (Δ*amr1*) were 2-fold greater on average than those made by the wild type or both complemented mutants (Δ*amr1:Amr1* and Δ*amr1:TrpCp-Amr1*) (Figure 2E and Table 4).

**Table 3:** Increased virulence of four strains of Δ*amr1* deletion mutants compared to wild-type *Alternaria brassicicola*.

Mutants	Plant age	d.f.	Lesion diameter (mm)		Increase of lesion diameter	*p*-value
			Wild type	Mutant		
Δ*amr1-1*	unknown	11	12.4±3.4	18.4±1.9	49%	1.78E-05
Δ*amr1-4*	7 weeks	11	9.7±8.0	17.7±6.6	82%	4.20E-04
Δamr1-4*	7 weeks	11	9.4±7.4	17.0±5.6	82%	1.40E-04
Δ*amr1-4*	5 weeks	5	9.0±3.3	12.0±2.5	30%	3.80E-02
Δ*amr1-5*	unknown	8	6.9±4.6	10.9±7.4	58%	1.80E-02
Δ*amr1:Amr1p-GFP*	5 weeks	5	8.0±2.8	12.0±2.3	28%	2.60E-02
Δamr1:Amr1p-GFP*	5 weeks	15	10.0±4.7	13.0±4.0	31%	3.00E-04

d.f. = degrees of freedom, P = probability, lesion size indicates the average lesion diameter. Results marked with an asterisk (*) were from pathogenicity assays with whole plants, others were from assays with detached leaves.

**Table 4:** Effect of the *Amr1* gene on lesion diameters (mm) following inoculation with wild-type *Alternaria brassicicola*, Δ*amr1:Amr1p-GFP*, Δ*amr1:Amr1*,and Δ*amr1:TrpCp-Amr1*.

Leaf number counted from lowest leaf	combined (N = 20)	wild type (N = 5)	Δ*amr1* (N = 5)	Compl (N = 5)	Cons (N = 5)	Virulence increase (%)
						Δ*amr1 vs* .wild type
5 (old)	8.5±4.0^a^	7.6±3.0	14.8±2.9	8.6±1.9	7.2±1.9	137±122
6	8.7±4.0^a^	7.8±3.4	14.4±1.3	9.4±3.4	6.8±3.6	127±125
7	8.6±3.7^a^	8.0±2.8	14.2±1.6	7.6±3.2	7.4±2.6	95±65
8 (young)	6.5±3.7^b^	4.4±1.8	13.0±0.7	5.2±1.7	5.4±1.9	227±111
Mean		7.0±3.1^B^	14.1±1.8^A^	7.7±3.1^B^	6.7±2.5^B^	
Pr>F	0.0001**	0.0395*	0.1615	0.0489*	0.0028**	

Mean lesion diameters (mm) and their standard deviations in each column followed by the same letter were not significantly different based on the Waller-Duncan *k*-ratio (*k* = 100) t-test. * and ** indicate a significant effect of leaf age based on analysis of variance at *p*<0.05 and *p*<0.01, respectively. Increased virulence was calculated by the sum of lesion size differences between the wild type and mutant divided by the sum of wild-type lesion sizes multiplied by 100. combined = all data with lesions caused by wild type, Δ*amr1*, Comp, and Cons mutants. Δ*amr1 = Δamr1:Amr1p-GFP*, Compl = Δ*amr1:Amr1*, Cons = Δ*amr1:TrpCp-Amr1.*

We performed additional pathogenicity assays using as few as ∼300 spores to test the effect of low inoculum concentration on pathogenesis (Figure 2 B, C). Lesions caused by ∼440 wild-type conidia were small and some stopped expanding, while lesion diameters caused by ∼400 Δ*amr1:Amr1p-GFP* mutant conidia were on average ∼3-fold larger (*p*<0.0001, *df* = 29, two tailed *t*-test) and continued to expand throughout the infection process. Inoculation with approximately ∼330 wild-type conidia produced black spots, indicating successful penetration but a failure to colonize host tissue. Lesion diameters created by the same concentration of mutant conidia, however, were on average ∼5-fold larger (*p*<0.0001, *df* = 29, two tailed *t*-test) and continued to expand during the five-day experiments. This result demonstrated that the Δ*amr1* mutants required fewer conidia than the wild type for successful infection.

### *Amr1* gene expression and its protein localization during plant infection

**Figure 3:**
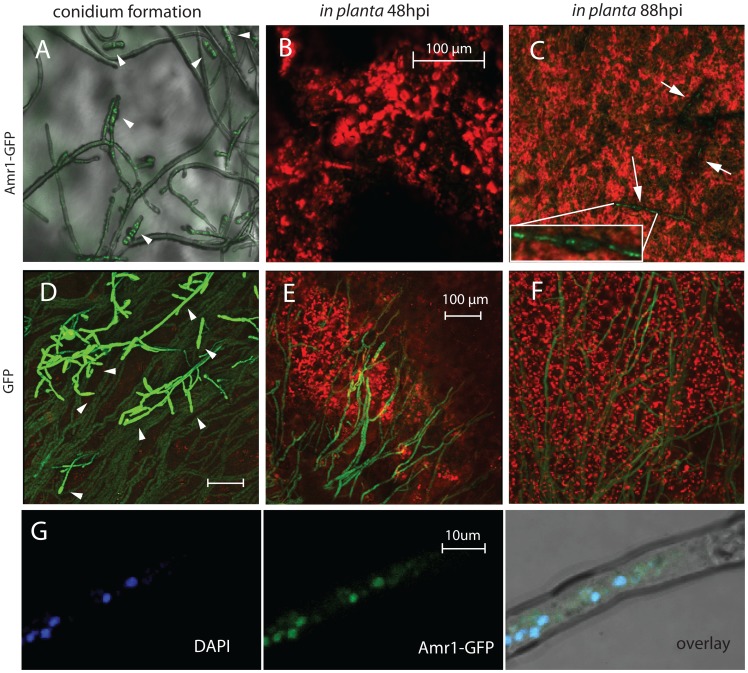
Confocal microscopic images showing *Amr1* gene expression and its protein localization in fungal nuclei during conidiation and pathogenesis. A–C. Green fluorescence indirectly shows localization of Amr1 protein in fungal nuclei: GFP is expressed as the Amr1-GFP fusion protein. D–F. Green fluorescence in fungal tissues indicates an abundance of GFP, whose gene is under the regulation of the *Amr1* promoter in the Δ*amr1:Amr1p-GFP* mutant. G. Fluorescence shows co-localization of Amr1-GFP proteins, and 4′,6-diamidino-2-phenylindole (DAPI) stains that bind to nucleotides. Pink color represents auto-fluorescence of plant tissues. Arrowheads indicate conidia. Arrows point to fungal hyphae with localized green fluorescence in the nuclei during late plant infection. hpi =  hours post-inoculation.

In order to support the notion that AMR1 is a transcription factor, we monitored the localization of AMR1 protein using a mutant strain expressing an AMR1-GFP fusion protein. The AMR1-GFP fusion protein was primarily located in the nuclei of highly melanized conidia and aerial hyphae (Figure 3A). AMR1 protein expression in conidia was consistent with its proposed regulatory role in melanin biosynthesis during conidiogenesis in *B. oryzae*, *Coc. heterostrophus*, *Col. lagenarium*, and *M. grisea* [21], [22], [41]. We could not detect the fluorescent fusion protein in hyphae growing on PDA (data not shown) or at 24 hours post-inoculation (hpi) *in planta* during the early stages of pathogenesis (Figure 3B). In contrast, the fusion proteins were detected in the nuclei of hyphae actively invading host tissues during the late stage of infection (Figure 3C). As a negative control, expression and localization of GFP were also monitored in a promoter-tagged mutant (Δ*amr1:Amr1p-GFP*) in which GFP expression was regulated by the native *Amr1* promoter (Figure 3 D–F). GFP in the promoter-tagged mutant was distributed throughout the cytoplasm of all fungal tissues, including conidia. The GFP continued to be expressed at moderate levels in the hyphae of the Δ*amr1:Amr1p-GFP* mutant, even during the early stages of plant infection when AMR1-GFP protein was undetectable (Figure 3 B, E).

### Melanin deficiency and pink pigment secretion by mutants

**Figure 4:**
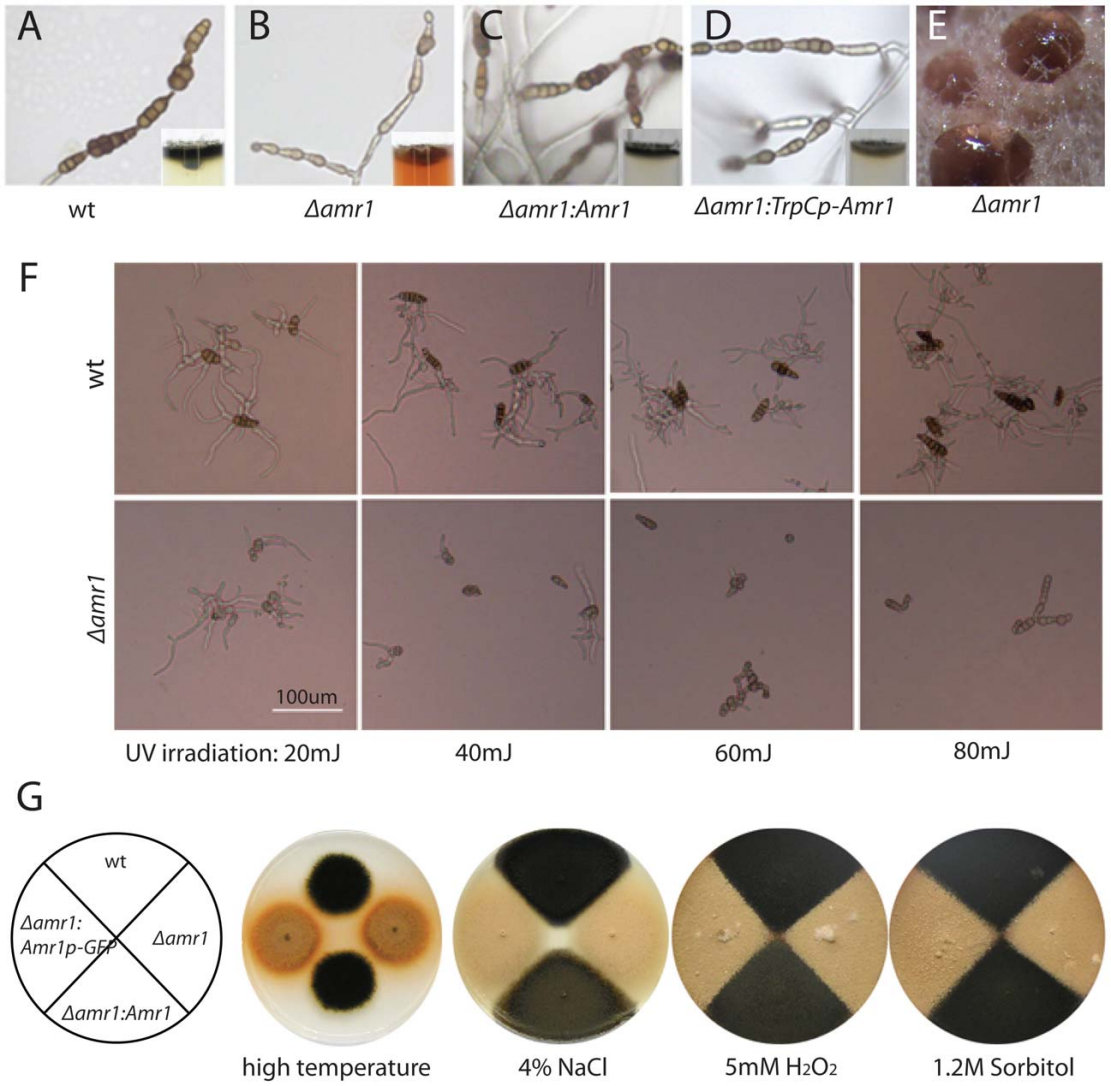
Melanin deficiency and its effects on fungal morphology and responses to stressors. A–D. Conidial chains produced by each strain. Insets: fungal growth of each strain showing colony color and pink pigment secreted by Δ*amr1* mutants during saprophytic growth on PDA. E. Pink exudate from the tips of conidial chains of the Δ*amr1* mutant. F. Germination and hyphal growth after UV irradiation. G. Growth comparisons between mutants and wild type either under high temperature (33°C), or in the presence of the indicated chemical. Abbreviations: Δ*amr1* = *Amr1* deletion mutant; Δ*amr1:Amr1* = Δ*amr1* mutant complemented with the *Amr1* allele; Δ*amr1:TrpCp-Amr1* = mutant that constitutively expresses the *Amr1* gene under control of the *TrpC* promoter.

All strains of verified gene disruption and deletion mutants (*amr1*, Δ*amr1*, and Δ*amr1:Amr1p-GFP*) produced melanin-deficient conidia. The color of the mutant colonies, however, was orange by 3 days post-inoculation (dpi) when grown on a PDA medium (inset in Figure 4 A, B). The orange color became denser with increased conidia production, or when the culture was exposed to light. The pigment was not accumulated in fungal tissues but was secreted into the medium. Droplets of pink pigment were visible at the tips of many conidial chains on PDA and on plant surfaces by 7 dpi (Figure 4E). The pigment was also secreted during the culture in liquid medium (Figure S3A). The pigment did not have phytotoxic effects on host plants (Figure S3B). Furthermore, addition of pink pigment to inocula of wild-type conidia did not affect lesion size (Figure S3C). The wild-type allele of the *Amr1* gene, controlled by its own promoter or by the *TrpC* promoter, restored the ability of the Δ*amr1* mutants to accumulate melanin (Figure 4 C, D) and pink pigment was no longer visible. Conidia produced by the mutant complemented with *TrpCp-Amr1* constructs were slightly lighter than the taupe-colored wild-type conidia. None of the complemented mutants secreted pink pigment to the medium.

### Susceptibility of Δ*amr1* mutants to abiotic stress

Because melanin plays an important role in protecting fungi against various types of stress, we examined the effect of UV irradiation, glucanase, heat, salt, and osmolites on Δ*amr1* mutants. UV irradiation at 80 mJ had a marginal effect on conidial germination and hyphal growth of wild-type *A. brassicicola* (Figure 4F, upper panel). However, 40 mJ of UV irradiation severely affected conidial germination and hyphal growth of the mutants, and 60 mJ almost eliminated germination of the Δ*amr1*mutant (Figure 4F, lower panel). It took more than three hours for β-glucanase to digest the melanized cell walls of wild-type *A. brassicicola* spores, but less than one hour for it to digest the cell walls of the melanin-deficient Δ*amr1* mutant (data not shown).

Germination and vegetative growth were not significantly different between the wild type and Δ*amr1* mutants under high-temperature conditions or in the presence of NaCl, H_2_O_2_, or sorbitol. The colony size was comparable for both the Δ*amr1* and Δ*amr1:Amr1p-GFP* mutants, a complemented mutant, and the wild type on the same media (Figure 4G).

### Melanin deficiency and virulence of *abpks7* mutants

We produced gene disruption mutants of the melanin-associated polyketide synthase (*Pks*) homolog in *A. brassicicola* (*AbPks7*). The *Pks* uses acetyl coenzyme A or malonyl coenzyme A as a precursor and produces a melanin-intermediate product, 1,3,6,8-tetrahydroxynaphthalene (T4HN). The *abpks7* disruption mutants produced melanin-deficient conidia (Figure S4 A–C). These melanin-deficient mutants caused lesions comparable to the wild type on their host plant, *B. oleracea* (Figure 4D), further demonstrating that melanin is not a virulence-associated factor in this fungus.

### Enhanced growth of the Δ*amr1*mutant in media containing pectin

**Figure 5:**
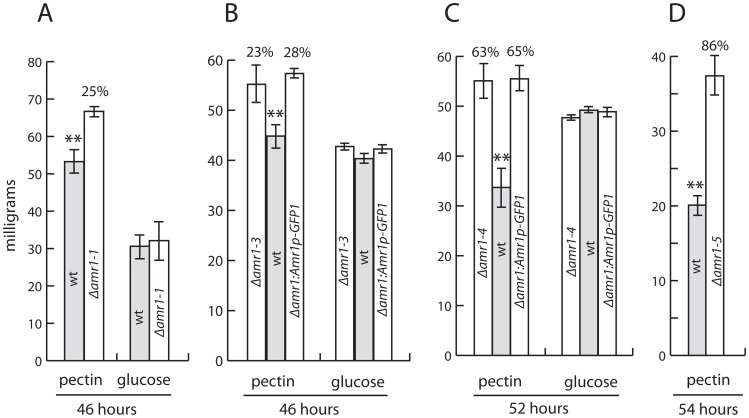
Effect of pectin on the vegetative growth of four strains of Δ*amr1* mutants and wild-type *Alternaria brassicicola*. A–D. Data show mean dry weight in milligrams. Each graph is the result of an independent experiment. The poor correlation between the absolute amount of dry biomass and the length of the incubation period was partially due to differences in the number of conidia used in each experiment. Values above the bars indicate the percent increase in dry biomass of the mutant compared to the wild type. Hours under each chart show the length of the incubation period. ** indicates *p*<0.01. Error bars represent standard deviation. wt = wild type.

Carbohydrate-active enzymes, such as glycoside hydrolases, polysaccharide lyases, and carbohydrate esterases are slightly expanded in *A. brassicicola* as has been found in other plant pathogenic Dothideomycetes [35]. The enzymes encoded by these genes are thought to be involved in the breakdown of complex carbohydrates in the cell walls of host plants. We indirectly evaluated the role of *Amr1* in regulating genes encoding putative cell wall-degrading enzymes by comparing the vegetative growth (dry weight of mycelium) of the Δ*amr1-1* and Δ*amr1-4* mutants to the wild type on various carbon sources. A minimal broth medium was supplemented with either 1% glucose, or the common cell wall polysaccharides α-cellulose, xylan, or pectin. Vegetative growth of the Δ*amr1-1* and Δ*amr1-4* mutants was the same as the wild type in the presence of glucose, xylan, lignin, and α-cellulose (data not shown). However, growth of these mutants was greater than the wild type in the presence of citrus pectin (Figure 5 A, C). To verify this observation, we tested the effects of pectin on the growth of the Δ*amr1-3*, Δ*amr1-5*, and Δ*amr1:Amr1p-GFP* mutants. These mutants also showed a significant increase in vegetative growth over the wild type in the presence of pectin (Figure 5 B, D). Vegetative growth was up to 86% greater than the wild type when mutants were cultured longer than 46 hours (Figure 5 C, D). There was no significant difference in growth between the wild type and the mutants in the presence of glucose. Because all Δ*amr1* mutants showed similar levels of melanin deficiency, pink pigment secretion, and pectin utilization, we used Δ*amr1-4* in subsequent gene expression profiling studies.

### Regulation of genes by *Amr1* during pathogenesis

**Figure 6:**
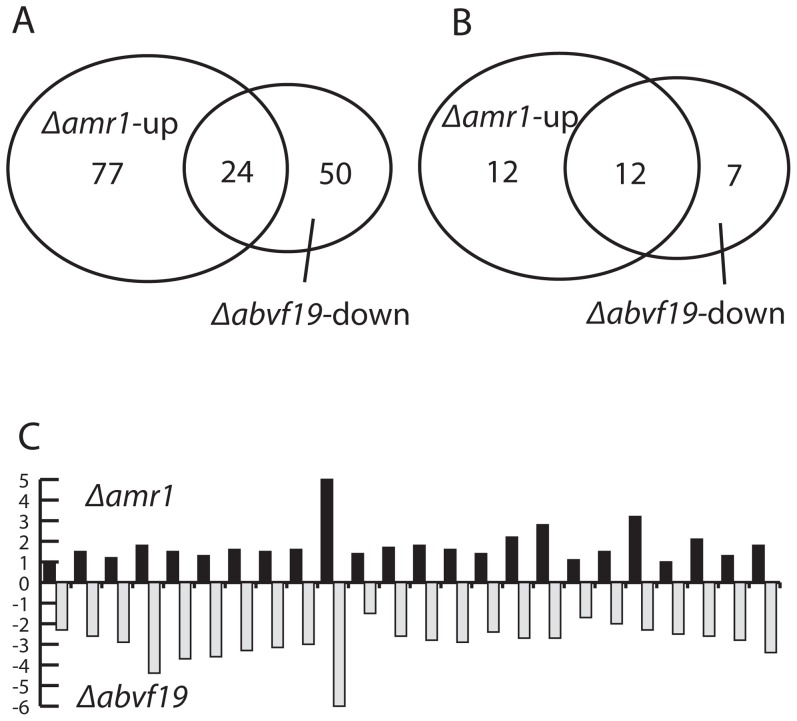
Comparison of differentially expressed genes in the Δ*amr1* and Δ*abvf19* mutants compared to wild-type *Alternaria brassicicola*. A. Comparison between up-regulated genes in the Δ*amr1* mutant and down-regulated genes in the Δ*abvf19* mutant during the late stage of infection. B. Number of differentially expressed glycoside hydrolase genes. C. Comparison of expression levels for the 24 genes differentially expressed in the Δ*amr1* and Δ*abvf19* mutants compared to the wild type. Y-axis shows Log_2_ (mutant)_expression_-Log_2_ (wild type)_expression_).

We used RNA-seq approach to determine the subset of genes regulated by *Amr1*. Using the RNA-seq approach, gene expression profiles were obtained and then compared between a Δ*amr1* mutant and the wild type during the late stage of infection on green cabbage. At this time, the AMR1 protein was localized in the nuclei of infecting hyphae. A total of 6.17×10^7^ reads were produced for the wild type and 6.94×10^7^ for the Δ*amr1* mutant. Of these, 4.14×10^7^ (67.1%) tags for the wild type and 4.78×10^7^ (68.9%) tags for the mutant were mapped to the genome of *A. brassicicola*. Of *A. brassicicola*'s 10,688 predicted genes, 101 and 163 genes were significantly (*p*<0.05) up- or down-regulated more than 2-fold, respectively, in the Δ*amr1* mutant compared to the wild type (Table S2). This respectively represents 0.95% and 1.53% of the genes in the *A. brassicicola* genome. Functional categories that were overrepresented in the up-regulated genes included putative cell-wall depolymerization enzymes (Table S3). They included 21 glycoside hydrolases, two pectate lyases, and one pectin esterase (Table S2). In addition, two cutinases and one lipase enzyme were also up-regulated in the mutant. One of the two cutinases, *CutAb*, was up-regulated >32-fold. The necrosis-inducing protein gene (3.1-fold), two P450 genes (2.8- and 2.6-fold), and the homolog of the peptidase aegerolysin (3.3-fold) were also up regulated in the mutant. 23 of 101 up-regulated genes in Δ*amr1* mutants were expressed at a lower level in the previously characterized Δ*abvf19* mutants and eleven of the shared genes between the two mutants were glycoside hydrolases (Table 2 and Figure 6). Genes in the melanin synthesis pathway were among the down-regulated genes. As expected, the expression level of *Amr1* was close to zero (background noise level) in the Δ*amr1*mutant during the late stage of infection, compared to a high level of induction in the wild type (Table S2). Expression levels of other structural genes in the melanin synthesis pathway*—Brn2*, *Scd1*, and *Brn1* (Eliahu *et al*., 2007; Tsuji *et al.*, 2000)*—*were 8- to 30-fold lower in the mutant than in the wild type. Differential expression between the wild type and Δ*amr1* mutant was verified by quantitative real time PCR (qRT-PCR) for seven differentially and three similarly expressed genes (Table S2). *AbPks7* was annotated as three separate genes and their expression in the mutants was reduced about four-fold (Table S2).

### Effect of *Amr1* on gene expression during the early infection stage

**Figure 7:**
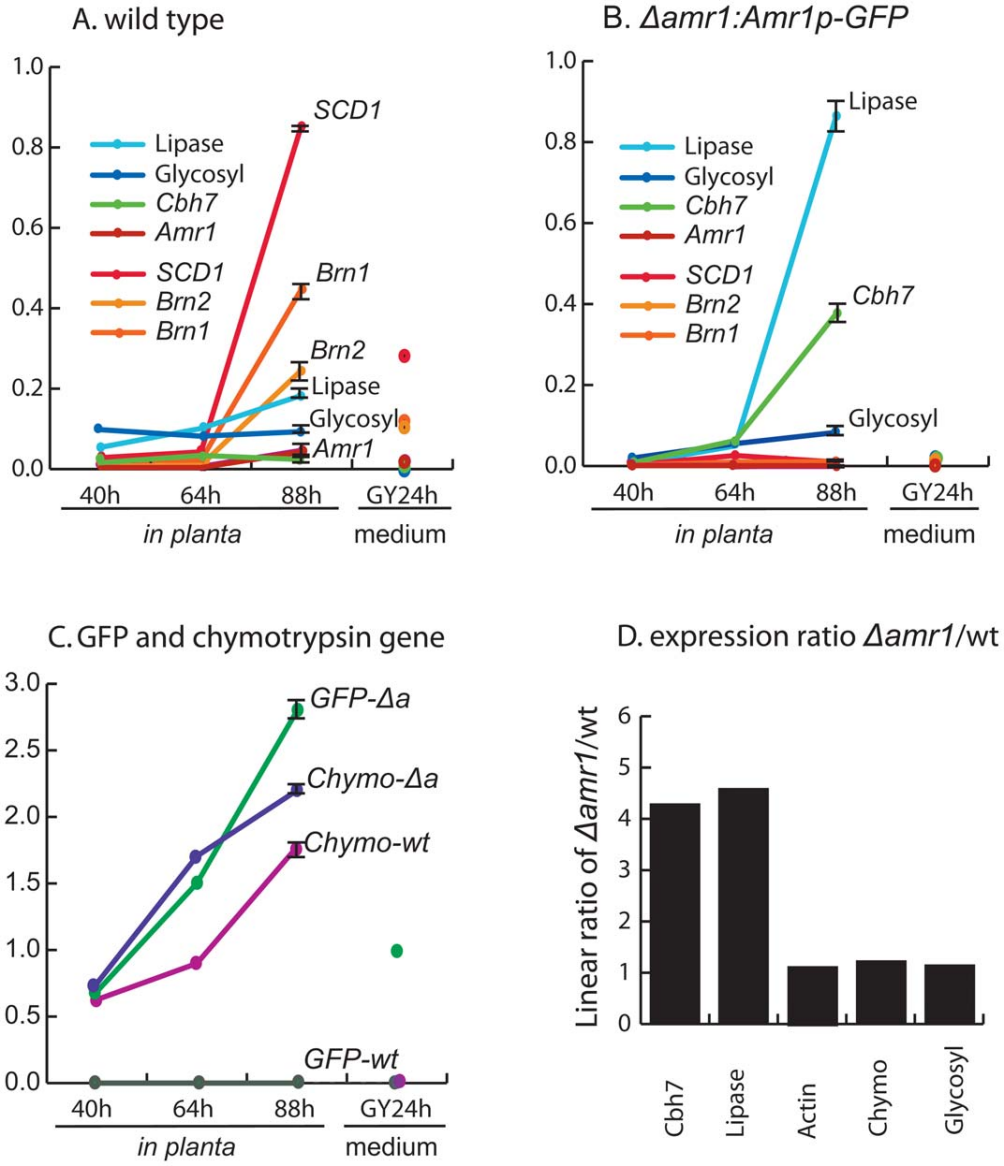
Expression of melanin biosynthesis-associated genes and four hydrolytic enzyme-coding genes. A–C. Relative transcription abundance of each gene was determined in comparison to actin gene transcripts in the same tissue. Y-axes show relative abundance of the transcripts compared to the actin gene. D. Expression ratio between the Δ*amr1* and wild type during late stage of infection. A total of three biological replicates (N = 3) were used for this study. Bars represent standard error. wt = wild type, Δ*a* = Δ*amr1:Amr1p-GFP*, GY = glucose yeast extract broth. *SCD1* = Scytalone dehydratase, *Brn1* = T3HN reductase, *Brn2* = T4HN reductase, *Cbh7* = cellobiohydrolase, *Amr1* = Alternaria melanin regulation, chymo = chymotrypsin.

We examined the expression of the nine genes at two additional time points when *Amr1* was expressed at a low level. As expected, the *Amr1* gene and its three downstream genes in the melanin synthesis pathway (*Brn2*, *Scd1*, and *Brn1*) were not expressed in the Δ*amr1:Amr1p-GFP* mutant under any of the tested conditions (Figure 7B). They were expressed at a low level in the wild type at 40 and 64 hpi, when *Amr1* expression was low. When the *Amr1* transcripts were slightly increased at 88 hpi, however, the three genes were highly induced in the wild type (Figure 7A). The expression level of *Amr1* was extremely low in the wild type compared to the expression of *GFP* in the Δ*amr1:Amr1p-GFP* mutant. This may explain why the gene expression level of GFP is high in the Δ*amr1:Amr1p-GFP* mutant, but AMR1-GFP fusion protein was not detected during early infection (Figure 3 B, E). Moreover, this could have been due to the absence of feedback inhibition from *Amr1*. It may also have been caused by the increased stability of *GFP* mRNA, its protein, or both as compared to *Amr1*. The high stability of GFP protein has previously been reported (reviewed in [40]).

The lipase and cellobiohydrolase *Cbh7* (AB06252.1) genes were weakly expressed in the wild type from early pathogenesis until 64 hpi; during this time the *Amr1* expression level was low in the wild type. There was no expression of these two genes during saprophytic growth in an axenic medium by either the wild type or the mutant (Figure 7 A, B). At 88 hpi, the time when *Amr1* was induced in the wild type, *Cbh7* and lipase induction were 4.3- and 4.7-times greater respectively (*p*<0.001, *t*-test, *df* = 2) in the Δ*amr1:Amr1p-GFP* mutant than in the wild type (Figure 7D). The glycoside hydrolases and chymotrypsin genes were not expressed in either the wild type or the mutant during saprophytic growth in glucose yeast extract broth (GYEB) (Figure 7 GY24h). Expression of the chymotrypsin gene was twice as high in the mutant at 64 hpi as in the wild type, but there was little difference in chymotrypsin levels at 88 hpi (Figure 7C). In summary, transcript levels of putative hydrolytic enzyme genes, including lipase and *Cbh7*, were moderately higher with statistical significance in the mutant than in the wild type during the colonization of host plants (Figure 7). This coincided with the time when the *Amr1* gene was induced in the wild type.

### The *AbVf8* gene is a pathogenesis-associated transcription factor

Another virulence factor identified in these studies was the putative transcription factor *AbVf8.* The *AbVf8* gene showed no sequence similarities to known pathogenesis-associated genes in any fungus. *AbVf8* in *A. brassicicola* and its homologs in other fungi (e.g., in *Pyrenophora* spp. or *Phaeosphaeria nodorum*) were annotated as either a hypothetical protein or a predicted transcription factor. The diameters of lesions caused by disruption mutants of *AbVf8* (*abvf8*) were 50–80% smaller than those produced by the wild type. Vegetative growth rates and the formation of conidia were comparable to the wild type. Predicted domains in the protein sequence of the *AbVf8* genes were somewhat informative regarding their functions. The predicted protein encoded by *AbVf8* contained a putative SET domain. SET domains are thought to be an epigenetic regulator of gene expression during development and modulate chromatin structure.

Because it was a novel transcription factor associated with pathogenesis and had a possible association with epigenetic regulation, we decided to confirm the results of pathogenicity assays using additional mutants. We made gene deletion mutants (Δ*abvf8*) by replacing the coding region with a HygB resistance cassette. Southern hybridization confirmed that the *AbVf8* gene was replaced by a single copy of the HygB resistance cassette in nine transformants (Figure S2). One transformant had an ectopic insertion of multiple copies of the HygB resistance cassette. Pathogenicity assays using Δ*abvf8-2*, Δ*abvf8-3*, and an ectopic insertion mutant produced results consistent to those we produced with the gene disruption mutants. Both deletion mutants caused lesions with diameters 55% smaller than lesions caused by the wild type or the ectopic insertion mutant (Table 2). We complemented the Δ*abvf8-2* mutant with the wild-type allele of the *AbVf8* gene. Complemented mutants restored its ability to produce lesions comparable to the wild type (data not shown). We will further characterize the functions of the *AbVf8* gene in the future.

## Discussion

### Transcription factors associated with pathogenesis

Of 138 *A. brassicicola* transcription factor genes with zinc finger domains and other regulatory genes analyzed to date in this and previous studies, only six genes were strongly associated with pathogenesis. These six genes were *AbSte12*, *AbPacC*, *AbVf19*, *Amr1*, *AbVf8*, and *AbPro1* (Table 1). This result was comparable to the comprehensive knockout study of 693 putative transcription factors in *Fusarium graminearum*, where 62 genes affected virulence [42]. *AbSte12* is a homolog of the *Ste12* gene with mutants that are either nonpathogenic or reduced in virulence in several fungi (reviewed in [43]). *Ste12* is regulated by a MAP kinase, *Fus3/Kss1*. Mutants of either homologous gene in *A. brassicicola* showed defective conidial development and were nonpathogenic [14], [15]. The *AbPacC* gene has a single copy in the genome sequence of *A. brassicicola* and shows a high sequence similarity (E = 0.0) with the *PacC* gene in other fungi. *PacC* is the transcription factor in the pH regulation pathway of *Aspergillus nidulans* [44]. It also controls the expression of fumonisin toxins in *F. verticillioides* [45] and endopolygalacturonase and oxalic acid in *S. sclerotiorum* [38], [46]. Mutation of its homologous genes reduced virulence in *S. sclerotiorum* and *Col. acutatum* and increased virulence in *F. oxysporum* [37], [38], [47]. *AbVf19* regulates a suite of genes that are important for decomposing and utilizing plant materials during the late stage of plant infection [20]. We have yet to characterize the functions of *AbVf8* and *AbPro1*. Regardless of their functions, however, all mutants studied to dates except *Amr1* were nonpathogenic or showed a reduction in virulence.

### Melanin biosynthesis and virulence in *A. brassicicola*

Melanin is a ubiquitous pigment that plays an important role in protecting fungi from the damaging effects of environmental stress. It accumulates in the cell walls of hyphae and conidia during the late stationary phase of mycelial growth. It also forms under stressful conditions, including ultraviolet irradiation, a hyperosmotic environment, nutrient deprivation, or an accumulation of toxic wastes during *in vitro* culture. Over-expression of a *Cmr1* ortholog in *Bipolaris oryzae* (*Bmr1*) caused continuous induction of the three downstream genes in the melanin synthesis pathway and increased melanization of the colonies [41]. The loss of function of *Cmr1*, its homologs, or their downstream genes in other fungi, resulted in melanin deficiency [21], [22], [25], [48]. We identified a transcription factor in this study, *Amr1*, which regulates the melanin synthesis pathway in *A. brassicicola*. The melanin synthesis-associated structural genes *Brn1* and *Pks* are located together in a 30 Kb region and their organization and orientation is the same as in the closely related fungus *Coc. heterostrophus* [21]. Interestingly, as reported here melanin was not associated with virulence in *A. brassicicola* as has been reported in other fungi [21]. However, based upon our data there appears to be a linkage between melanin biosynthesis and a reduction in virulence, perhaps due to a lifestyle switch from pathogenesis to reproduction.

### Mutation of the *Amr1* gene unexpectedly caused increased virulence

*Amr1* gene knockout mutants, both gene disruption (*amr1*) and gene deletion (Δ*amr1* and Δ*amr1:Amr1p-GFP*), were easily recognized by the lack of melanin in their conidia, orange-colored colonies, and the secretion of a pink pigment (Figure 4). These phenotypes were similar to those found in mutants of its homologs in other fungi [21], [22], [41]. The Δ*amr1*mutants were more susceptible to UV light and enzyme digestion (Figure 4). Unexpectedly, the *Amr1* gene knockout mutants were more virulent than the wild type, producing larger lesions (Table 3), and required fewer conidia for successful infection (Figure 2). Mutation of *Amr1* homologs in the phytopathogenic fungi *M. grisea* and *Col. lagenarium* produced melanin-deficient conidia. The appressoria of these mutants, however, remained melanized suggesting an alternate regulatory mechanism and pathogenicity was not affected [22]. Melanin deficiency caused by a mutation of other genes in the melanin biosynthesis pathway was associated with a loss of pathogenicity in *M. grisea* and *Col. lagenarium* [49], [50], [51] and a decrease in virulence in the opportunistic human fungal pathogens *Aspergillus fumigatus* and *Waggiella dermatitidi*s [52], [53]. In most other plant-pathogenic fungi, melanin deficiency usually had little effect on pathogenesis under controlled laboratory conditions [25], [31], [48], [54]. Melanin deficient mutants of *Coc. heterostrophus*, however, were nonpathogenic under field conditions possibly due to increased sensitivity to UV light outdoors [54]. In summary, the increased virulence of the Δ*amr1* mutants was unexpected and unique among melanin-deficient mutants of pathogenic fungi.

### The cause of increased virulence in Δ*amr1* mutants

The pink pigment secreted by Δ*amr1* mutants was neither toxic to host plants nor beneficial for the wild type *A. brasscicola* to infect host plants (Figure S3). Furthermore, melanin-deficient mutants of another gene, *abpks7*, showed no changes in virulence (Figure S4). These data suggested that neither the presence of pink pigment nor the lack of melanin is associated with the increased virulence of the Δ*amr1* mutants investigated in this study. Additionally, there was no difference in germination or vegetative growth between the Δ*amr1* mutants and the wild type in the presence of various chemicals (Figure 4G). However, the mutants were more susceptible than wild type to UV light (Figure 4F) and the cell wall-degrading enzyme, glucanase. These data suggested that the mutants' ability to respond to stress was not associated with their increased virulence, since plant glucanases are known to be defense enzymes.

The ability of the mutants to outgrow the wild type when citrus pectin was the major carbon source (Figure 5) suggested that the mutants were more efficient in utilizing the pectin component of plant cell walls and middle lamellae. The expression profile comparisons identified 30 hydrolytic enzyme genes expressed moderately more in the mutant than in wild type *A. brassicicola* during pathogenesis (Table S2). However, they included only two pectate lyase genes and one pectin esterase gene. Other genes upregulated in the mutant compared to the wild type included genes coding for two cutinases, a lipolytic enzyme and 20 other various glycoside hydrolases (GH). Interestingly, 6 of the 22 genes in the GH61-gene family were expressed at higher levels in the Δ*amr1* mutant. The GH61 family might have a role in degrading cellulose, lignocellulose, chitin, or other polysaccharides [55]. The GH61 family may be involved in digesting pectins whose side chains are structurally diverse in constituting sugars and glycosidic linkages. They may digest other cell-wall components, such as cellulose and hemicelluloses along with other glycoside hydrolases. It was not possible in this study to evaluate whether the Δ*amr1* mutants made better use of these cell wall materials because *A. brassicicola* grew poorly in the presence of α-cellulose, beechwood-xylan, and lignin. Our uninformative growth data was likely due to the non-brassica source of cell wall materials. Nonetheless, the gene expression and growth assay data indirectly support the importance of cell wall-degrading enzymes and possibly those involved in pectin digestion, since pectic polysaccharides constitute about one-third of the cell wall components and most of the middle lamella in dicotyledonous plants.

Induction of the *Amr1* gene in *A. brassicicola* did not occur until 64 hpi (Figure 7). Suppression of the *Amr1* gene might be important during pathogenesis when energy is allocated to colonization of host tissues and overcoming host defense mechanisms, rather than producing reproductive dispersal structures like conidia. In the wild type, the transcripts of hydrolytic enzyme genes were slightly increased compared to the actin transcripts, as previously reported [14], [56], [57]. In addition, when conidiation was initiated the induction of *Amr1* was accompanied by a sharp increase in transcripts of its downstream genes in the melanin synthesis pathway. There was also a small increase in the transcripts of hydrolytic enzyme-coding genes in the wild type. However, many of these genes, mostly expressing glycoside hydrolases, a few cutinases, and pectate digestion enzymes, were expressed at higher levels in the Δ*amr1-4* and Δ*amr1:Amr1p-GFP* mutants, which did not express *Amr1* (Figure 7D). This suggests that *Amr1* suppressed genes predicted to be involved in the digestion of lipids and carbohydrates. This occurred during late pathogenesis (88 hpi) when they were still needed to some extent for either killing [58] or digesting host tissue. Whether the *Amr1* gene is involved in the regulation of most hydrolytic enzymes sequentially induced in *A. brassicicola* during pathogenesis has yet to be investigated [24], [59]. Other genes encoding a necrosis-inducing protein, two P450s, the aegerolysin peptidase homolog, and other hypothetical proteins might have contributed to the increased virulence of the Δ*amr1* mutant and warrant further investigation. Interestingly, 24 genes expressed at a higher level in Δ*amr1* were expressed at lower levels in the Δ*abvf19* deletion mutants that displayed a reduction in virulence (Figure 6C), suggesting opposite roles of the two transcription factor genes in the regulation of pathogenesis-associated genes. This finding may aid in the selection of specific targets for future analyses of their functions in pathogenesis.

### Evolution of virulence in *A. brassicicola*

The melanin-deficient mutant phenotype and expression pattern of the *Amr1*, *Brn1*, *Brn2*, and *Scd1* genes in *A. brassicicola* suggested that the function of the *Amr1* gene was to regulate melanin synthesis during conidiogenesis. This function is highly conserved in other fungi [21], [22], [41]. In addition to regulating the melanin biosynthesis pathway, *Amr1* negatively regulated virulence. _ENREF_1The evolution of virulence has been a subject of extensive theoretical analysis. The “trade-off hypothesis” [60] produced a number of models showing a contested relationship between virulence and transmissibility. It challenges the hypothesis that strong virulence can seriously damage or kill host plants and threaten pathogen survival, so parasites will ultimately evolve to be less virulent. However, analyses of ecological and epidemiological factors related to virulence has produced inconsistent results [61]. Therefore, a modified trade-off theory predicted that strong parasites became moderately virulent to their hosts over time, but did not completely lose virulence [62]_ENREF_4. Our current work identified a transcription factor suppressing virulence but enhancing the ability of a fungus to preserve itself in space and time via melanin synthesis, primarily in conidia. This study provides an example of a transcription factor gene important for survival in nature that plays a critical role in negatively regulating virulence in the necrotrophic plant pathogen, *A. brassicicola.* The suppression of virulence we observed is unique and might have been acquired by this fungus, as this phenotype has not been observed in loss-of-function mutants of *Amr1* homologs in other species of plant pathogenic fungi. An ancestral fungal strain of *A. brassicicola* without the ability to suppress virulence by the activity of *Amr1* could have been a more virulent pathogen. Negative regulation of virulence is rare but exists in other phytopathogenic fungi. For example, a phosphatase enzyme in the MAP kinase signaling pathway negatively regulates virulence in *Ustilago maydis* [63]. Currently, *A. brassicicola* is a prolific saprophyte and necrotroph, efficiently colonizing susceptible, weakened, or dead host plants. Its pathogenicity, however, is inhibited on more resistant host plants. We speculate that the suppressive functions of *Amr1* contribute to the specialized adaptation of *A. brassicicola* as an efficient and successful facultative parasite.

## Materials and Methods

### Fungal strains, their transformation, maintenance, and primers

We used the facultative plant pathogen *Alternaria brassicicola* (Schweinitz, Wiltshire) (ATCC96836) in this study. Growth and maintenance of the fungus and its transformation, nucleic acid isolation, mutant purification, and mutant verification by Southern hybridization were performed as described previously [15]. The wild-type fungus and each of the mutant strains created during this study were purified by two rounds of single-spore isolation. The cultures were maintained as glycerol stock in separate tubes, with one tube used for each assay.

### Data availability

The genome sequence and gene predictions for *A. brassicicola* ATCC 96836 were obtained from http://genome.wustl.edu/genomes/view/alternaria_brassicicola/ and are available from DDBJ/EMBL/GenBank under accession ACIW00000000. In addition, the annotated genome is available in the context of other Dothideomycetes through the interactive JGI fungal portal MycoCosm [34] at http://jgi.doe.gov/Abrassicicola.

### Identification of putative transcription factors

The partially assembled genome sequence was annotated using an EnsEMBL annotation pipeline [64]. The HMM search results of FGENESH predicted peptides against PFAM and TIGRFAM databases were stored in a SQL database. A total of 251 PFAM accession numbers and 13 keywords descriptions were used to manually search for the putative transcription factors using SQL queries. Subsequently, the predicted amino acid, predicted cDNA, and genomic DNA sequences were manually retrieved from the database.

### Primer design and generation of targeted gene mutants

Four primers for targeted gene disruption and six primers for targeted gene deletion were synthesized for each gene (Table S4). We followed methods described previously for all steps from producing transformation constructs to verifying purified mutants [15], [65]. Briefly, constructs of up to 12 genes were independently transformed at a time. The transformed protoplasts were plated on a molten regeneration medium (1 M sucrose, 0.5% yeast extract, 0.5% casamino acids, and 1% agar) for 24 hours, followed by an overlay with the same volume of PDA containing 30 ug/ml HygB. On the seventh day after the transformation, four transformants of each gene were transferred to PDA plates containing 30 ug/ml HygB and cultured for five days. The transformants were purified by two rounds of single-spore isolation and verified by PCR using two sets of verification primers for each gene (Table S5).

### Generation of the *Amr1* deletion mutants, Δ*amr1* and Δ*amr1:Amr1p-GFP*

**Figure 8:**
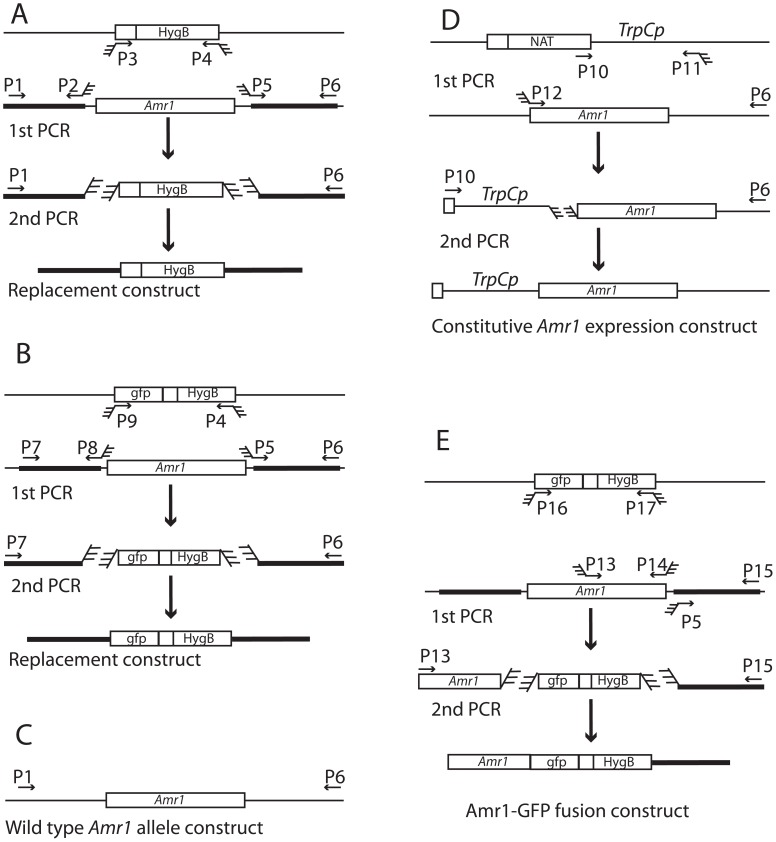
Schematic diagram of the PCR strategy used to make each construct. A. Construct for replacement of the *Amr1* gene with a Hygromycin B resistance cassette. B. Construct for replacement of the *Amr1* gene with a GFP coding region and Hygromycin B resistance cassette. C. Amplification of the wild-type allele of the *Amr1* gene. D. Construct for the constitutive *Amr1* expression cassette. E. Construct for the Amr1-GFP fusion protein expression.

All transformation constructs described in this work were produced by a double-jointed PCR method [66] with modifications described previously [15]. We made *Amr1* deletion mutants by replacing the gene with a HygB resistance cassette. The replacement construct was produced with the following three sets of primers (Figure 8). The primer set P1 and P2, and the primer set P5 and P6 were used to respectively amplify the 5′ and 3′ flanking regions of the targeted locus. Another set of primers, P3 and P4, was used to amplify the HygB-selectable marker gene cassette (1,436 bp) from pCB1636. In order to create mutants expressing green fluorescence under the control of the *Amr1* gene promoter, the *Amr1* promoter region and 3′ flanking region were amplified with P7 and P8, and P5 and P6, respectively. Another set of primers, P9 and P4 was used to amplify the 2,423 bp that covered the coding regions of the GFP and the HygB resistance cassette. All primers used Amr1gene are listed in Table S6.

### Complementation of Δ*amr1* mutants

The Δ*amr1-5* mutant was complemented with either the wild-type *Amr1* allele with its native promoter, or a chimeric construct of an *Amr1* allele under the control of a constitutive promoter. This promoter was derived from a *TrpC* gene (*TrpC*p) [67]. We used two primers, P1 and P6, to make constructs and reintroduce wild-type *Amr1* into the Δ*amr1* mutant. These primers amplified the 6,196 bp wild-type allele of the *Amr1* gene using *A. brassicicola* genomic DNA as a template. The PCR product included a 1,342 bp 5′ flanking region, 3,282 bp complete coding region, and a 1,570 bp 3′ flanking region. Separately, P18 and P19 were used to amplify a 2,226 bp-long nourseothricin-resistant cassette as a selectable marker gene, using a pNR vector as the template [68]. These two products (8 µg of *Amr1* and 7 µg of *NTC* cassette) were mixed to transform the Δ*amr1* mutant.

To produce constitutive *Amr1* expression mutants, three sets of primers were used to replace the 1,840 bp 5′ upstream to the predicted start codon with the *TrpC* promoter, *TrpC*p. We used two primers, P10 and P11, to amplify the 943 bp constitutive promoter region that includes a 152 bp partial coding sequence of NAT, 458 bp nonfunctional *ToxA* promoter, and a 333 bp-long functional *TrpC* promoter. Another set of promoters, P12 and P6, were used to amplify the 4,852 bp wild-type allele that spans the 3,282 bp coding region from the start codon to the stop codon of the *Amr1* gene and the 1,570 bp 3′ flanking region of the gene. The PCR products were mixed and diluted 10 times. The final mixed PCR products were used as template DNA to create *TrpCp-Amr1* gene constructs using two primers, P7 and P6. A total of 8 µg of the TrpCp-AMR1 chimeric gene constructs and 7 µg of the 2,226 bp-long nourseothricin-resistant cassette were transformed into the Δ*amr1* mutant. Among the nourseothricin-resistant transformants, two clones of the former (Δ*amr1:Amr1*) and four clones of the latter (Δ*amr1:TrpCp-Amr1*) were purified by two rounds of single-spore isolation in the presence of nourseothricin. Complementation of the mutant by a single copy of the *Amr1* gene was confirmed by Southern blot hybridization. All mutant clones were tested for purity on PDA by checking their growth patterns, growth rates, and uniformity of colony colors (sectoring) in the presence and absence of selectable markers.

### Generation of mutants expressing *Amr1-GFP* fusion proteins

In order to create mutants expressing green fluorescence protein fused to the C-terminal of AMR1, the *Amr1* coding region (1,209 bp) and 3′ flanking region (192 bp) were amplified with P13 and P14, and P5 and P15, respectively. Another set of primers, P16 and P17, was used to amplify the 2,384 bp that covered the coding regions of the GFP and the HygB resistance cassette. The final transformation constructs were produced by PCR amplification from the mixture of the PCR products using P1′ and 6′z9219-3R.

### Pathogenicity assays

Pathogenicity assays were performed with modifications as described previously using detached leaves harvested from 5- to 8-week-old plants or on the leaves of whole plants [15]. Commercially available seeds (Jonny's, Winslow, ME) of green cabbage (*Brassica oleracea*) or *Arabidopsis thaliana* were planted and grown under the same conditions for each pathogenicity assay. We grew green cabbage under 14 hours light 10 hours dark cycle and Arabidopsis under 10 hours light 14 hours dark cycle. Plants of similar heights and with similar-sized leaves were selected for each assay. Leaves were detached and placed in mini-moist chambers and randomly arranged on a laboratory bench for most assays. For the pathogenicity assays on whole plants, potted plants were placed in a semi-transparent plastic trough with adequate water. The troughs and plants were sealed with plastic wrap after inoculation to keep the relative humidity close to 100%. The increased virulence of each mutant was calculated using the formula (∑(Dm*i*-Dw*i*)/∑(Dw*i*))×100, where Dw*i* was the lesion diameter created by the wild-type for the *i*th sample and Dm*i* was the lesion diameter produced by the mutant for the *i*th sample (Table 2). We also analyzed lesion sizes among the wild type and various mutants (Tables 2). Lesion sizes were subjected to 5×4 (genotype×leaf position (leaf age)) two-way analysis of variance (ANOVA) using the general linear model (GLM) procedure in the Statistical Analysis System (SAS Institute, Cary, NC). Means were separated by the Waller-Duncan *k*-ratio (*k* = 100) *t*-test, or an *f*-test, whichever was appropriate. The Waller-Duncan test was chosen to minimize the Bayesian risk of the additive loss function.

### Confocal microscopy

Infected plant tissues were trimmed with a razor blade, placed on microscope slides, and covered with Gold Seal cover glasses. Confocal images were acquired using a 633 C-Apochromat (numerical aperture 1.2) water-immersion objective lens and an Olympus Fluoview 1000 Laser Scanning Confocal System on a IX-81 inverted microscope. Spectra for fungal tissues expressing standard green fluorescence and for plant cells emitting autofluorescence were collected by simultaneous 488 nm and 543 nm excitation using 30 mW argon and 1 mW helium∶neon lasers, respectively. The standard GFP spectrum was collected through 488 nm excitation using a 20 nm window from 505 to 525 nm. Plant tissue, including chloroplasts, was visualized using 543 nm excitation with a 560 nm-long pass filter. Images of fungal tissue grown in nutrient media were captured with 488 nm excitation and DIC-transmitted light. All fluorescent images were composed of multiple layers acquired with the Confocal System.

### Measuring the response to stressors

Each fungal strain from glycerol stocks was inoculated on PDA with an appropriate selectable agent and grown in the dark for 5 days at 25°C. In order to test for sensitivity to osmotic stress and oxygen radicals, wild-type and mutant conidia were pipetted onto PDA containing 2% (w/v) or 4% (w/v) NaCl, 0.6 M or 1.2 M sorbitol, or 2.5 mM or 5 mM H_2_O_2_. Mutant and wild-type strains were also cultured at 28, 30, and 33°C to measure the effect of temperature on their growth. Colony diameters were measured 4 days post-inoculation (dpi). To study the effect of UV light on germination, 1,000 spores were pipetted onto PDA, irradiated at 20, 40, 60, and 80 millijoules in a UV crosslinker (Agilent Technologies, Inc., CA), and incubated at 25°C for 24 hours. All experiments were conducted three times.

### Growth assays in the presence of sole carbon sources

Flasks (250 ml) containing a 50 ml broth of 0.5% (NH_4_)_2_SO_4_, 0.05% yeast extract, 0.15% KH_2_PO_4_, 0.06% MgSO_4_, 0.06% CaCl_2_, 0.0005% FeSO_4_ 7H_2_O, 0.00016% MnSO_4_ H_2_O, 0.00014% ZnSO_4_ 7H_2_O, and 0.00037% CoCl_2_ were supplemented with either 1% glucose or 1% of α-cellulose (cat #, C8002-1KG), or xylan (cat #, X4252-100G), lignin (cat # 471003-100G), or citrus pectin (cat #, P9135-500G) purchased from Sigma (St. Louis, MO). Each flask was inoculated with 4–6×10^5^ conidia of either Δ*amr1-1*, Δ*amr1-3*, Δ*amr1-4*, Δ*amr1-5*, Δ*amr1:Amr1p-GFP*, or the wild type. The flasks were incubated in the dark at 25°C with continuous agitation at 100 rpm. The flasks were shaken vigorously by hand several times during the first eight hours to prevent the conidia from sticking to the flask. Mycelia were harvested at 4 dpi, washed with distilled water, and dried at 70°C overnight. The increased biomass of each mutant and the wild type was calculated using the formula (∑(Wm*i*-Ww*i*)/∑(Ww*i*))×100, where Ww*i* was the dry weight of the wild type for the *i*th sample and Wm*i* was the weight of the mutant for the *i*th sample.

### Gene expression analysis by RNA-seq

To study the regulatory roles of *Amr1* in *A. brassicicola*, we compared gene expression profiles between the wild type and a Δ*amr1* mutant at 88 hours post-inoculation (hpi), a late stage of infection. We inoculated nine detached leaves harvested from three plants with conidia from the Δ*amr1-4* mutant and the wild type. Tissue samples containing both host plant tissue and fungal mycelium were harvested. Three biological replicates were produced for both the mutant and the wild type. Total RNA was purified from the tissue using an RNeasy kit (Quiagen, Palo Alto, CA). We used 4 µg of total RNA for each RNA sample to construct strand-specific sequencing libraries with a TruSeq RNA Sample Prep Kit (Illumina, San Diego, CA). Each library was constructed with unique index primers and all were run in a single lane. They were later decoupled using index primer sequences.

Sequence tags were mapped to the genome sequence of *A. brassicicola* using the programs TopHat 1.3.1 [69] and Bowtie 0.12.7 [70]. Default settings were used, except the segment length was set at 25 nucleotides and the number of allowed segment mismatches was set at 1 nucleotide. Additionally, intron length was designated as a minimum of 10 nucleotides and a maximum of 400 nucleotides. The program Cuffdiff, version 1.0.3, which is part of Cufflinks [71], was used to identify reads overlapping with previously predicted genes. The expression levels of each predicted gene were determined and normalized by the mapped Fragments Per Kilobase of exon model per Million (FPKM). Differentially expressed genes between the wild type and the mutant were determined by comparing FPKMs from three biological replicates for both the wild type and the mutant. We also applied a cutoff of at least a two-fold change in expression value for differential expression. The bias correction method was used while running Cuffdiff [72]. Custom scripts were written in Python to analyze the data.

### Representation analysis

Custom scripts were developed in Python and R to analyze over- and under-representation of functional annotation terms in sets of differentially regulated genes using the Fisher Exact test. The Benjamini-Hochberg correction was used to correct for multiple testing using a *p*-value of <0.05.

### Quantitative real-time PCR

Full-length sequences of the three downstream genes in the melanin synthesis pathway regulated by *Amr1* were identified in the *A. brassicicola* genome by Blast search. Primer sequences for each gene are listed in Table S6. The Δ*amr1:Amr1p-GFP* mutant and the wild type were used for qRT-PCR with three biological replicates of infected plant tissues. Each biological sample was collected from three or four leaves and total RNA was extracted using an RNeasy kit with DNAse digestion according to the manufacturer's protocol (Qiagen, Valencia, CA). Two micrograms of total RNA were transcribed to cDNA in a final volume of 20 µl using 50 ng of random pentamers and 200 ng of poly(T)_20_N with Superscript III (Invitrogen, Carlsbad, CA). Each cDNA was diluted 1∶20. Subsequent qRT-PCR reactions were performed in a 20 µl volume containing 120 nM of each primer, 1 µl of diluted cDNA, and 10 µl of FastStart SYBRGreen Master (Roche, Mannheim, Germany). Each reaction was run in a Biorad I-cycler (Bio-Rad, Hercules, CA, USA) as described previously (Cho *et al*, 2006). Relative amounts of the transcript of each gene were calculated as 2^−ΔCt^ using a threshold cycle (Ct), where ΔCt = (Ct,_gene*i*_ –Ct,_actin_). Fold changes of each gene between the wild type and Δ*amr1* strain were calculated as 2^−ΔΔCt^, where ΔΔCt = (Ct,_gene*i*_ –Ct,_actin_)*_Δamr1_ –* (Ct,_gene*i*_ –Ct,_actin_)_wild type_.

### Accession numbers

RNA-seq data: GEO Series Accession No. GSE36781 http://www.ncbi.nlm.nih.gov/geo/info/linking.html

*Amr1* (Alternaria melanin regulation) GenBank accession number: JF487829

## Supporting Information

Figure S1 **Verification of targeted gene disruption by polymerase chain reaction (PCR).** PCR amplification was performed with two pairs of target gene specific and HygB resistance gene primers. Relative primer binding locations are marked below the schematic diagram of the disrupted target gene on the left side of the gel images at the top row of page one. The diagrams were drawn based on a hypothesis that the target gene was disrupted by a single homologous recombination with a circularized linear minimal element construct as described previously (Cho Y, Davis JW, Kim KH, Wang J, Sun QH, et al. (2006) A high throughput targeted gene disruption method for *Alternaria brassicicola* functional genomics using linear minimal element (LME) constructs. Mol Plant Microbe Interact 19: 7–15.). Hatched box = a selectable marker gene, white box = a target genomic locus, and gray box = partial target gene. Amplification was performed with Taq polymerase for PCR reactions (Invitrogen, Carlsbad, CA, U.S.A.).
(DOC)

Figure S2 **Creation of** **Δ*abvf8* deletion mutants and pathogenicity assays.** A. Schematic diagram of the wild-type locus of the *AbVf8* gene, a replacement construct, and a mutant locus. The mutant locus depicts incorporation of the replacement construct into a wild-type locus by double homologous recombination resulting in gene replacement. B. Replacement of the *AbVf8* coding region with the selectable marker, Hygromycin B (HygB) resistance cassette. Absence of a wild-type band in nine lanes on the *AbVf8*-probe blot (above) indicates loss of the targeted gene. On the HygB-probed blot (below), the expected 4.6 Kb band indicates a single-copy insertion of the Hyg B resistance cassette in 9 lanes. Asterisk (*) indicates lanes of mutants used in this study. Probe regions are marked by *p-HygB* and *p-AbVf8*. C. Lesions on *Brassica oleracea* 5 days after inoculation with ∼2,000 conidia of the wild type (wt), two Δ*abvf8* mutants, and an ectopic insertion mutant. Abbreviations: H = *Hin*d III enzyme digestion site, wt = wild type *Alternaria brassicicola*, Ect = ectopic insertion mutant.
(DOC)

Figure S3 **Pigment secreted by Δ*amr1* mutants and its effect on pathogenesis and host plants.** A. Mycelia of the Δ*amr1* mutant at the bottom of the flask (left) is melanin deficient compared to the dark-colored wild-type mycelia (right). Both were cultured in 1% glucose and 0.5% yeast extract broth (GYEB) for three days, in the dark, with continuous agitation at 100 rpm. B. No toxic effects from the pigment. The pigment in 10 ml of GYEB was extracted with 10 ml chloroform, twice. The chloroform was removed by evaporation and the pigment dissolved in 1 ml of sterile water. The concentrated pigment was injected into green cabbage leaves to evaluate its toxic effect. Both the pigment and negative control (water) produced similar small water-soaked areas that disappeared within 24 hours. Injected areas are marked with circles. C. No visible effect of the pigment on plant infection. Wild-type conidia were inoculated either in water (left) or in pigment (right). Lesion size was not significantly different between the two treatments.
(DOC)

Figure S4 **Comparison of phenotypes between** ***abpks7*** **mutants and wild-type** ***Alternaria brassicicola*** **.** A. Stereoscopic view of an *abpks7* mutant colony showing melanin-deficient aerial hyphae and conidia. B. Stereoscopic view of a wild-type colony showing melanized aerial hyphae and conidia. C. Hyaline conidia of an *abpks7* mutant. Mutant conidia were mixed with a small number of dark, wild-type conidia to illustrate the color difference. D. Pathogenicity assay showing comparable lesions caused by three *abpks7* mutants and wild-type *A. brassicicola*. Average lesion diameters caused by the mutants and wild type were not statistically different.
(DOC)

Table S1 **List of transcription factor genes whose mutants were screened in this study.**
(XLS)

Table S2 **List of genes differentially expressed in the Δ** ***amr1*** **mutants compared to the wild type.**
(XLS)

Table S3 **Statistically over-represented functional annotation terms among 101 up-regulated genes in the** **Δ*amr1* mutant compared to the wild type.** The 101 genes were selected based on the statistical analysis included in Cufflink with at least a two-fold-difference.
(DOC)

Table S4 **Disruption Primers (5′ to 3′ direction).**
(DOC)

Table S5 **PCR verification primers (5′ to 3′ direction).**
(DOC)

Table S6 **List of primers used for qRT-PCR and transformation constructs.**
(DOC)
