# Supplementary material for: Transcription Factor Amr1 Induces Melanin Biosynthesis and Suppresses Virulence in Alternaria brassicicola
Source: PLoS Pathog. 2012 Oct 25;8(10):e1002974. doi: 10.1371/journal.ppat.1002974 (PMC3486909; doi:10.1371/journal.ppat.1002974)

Figure S1. Verification of targeted gene disruption by polymerase chain reaction (PCR). PCR amplification was performed with two pairs of target gene specific and HygB resistance gene primers. Relative primer binding locations are marked below the schematic diagram of the disrupted target gene on the left side of the gel images at the top row of page one. The diagrams were drawn based on a hypothesis that the target gene was disrupted by a single homologous recombination with a circularized linear minimal element construct as described previously (Cho Y, Davis JW, Kim KH, Wang J, Sun QH, et al. (2006) A high throughput targeted gene disruption method for *Alternaria brassicicola* functional genomics using linear minimal element (LME) constructs. Mol Plant Microbe Interact 19: 7-15.). Hatched box = a selectable marker gene, white box = a target genomic locus, and gray box = partial target gene. Amplification was performed with Taq polymerase for PCR reactions (Invitrogen, Carlsbad, CA, U.S.A.).


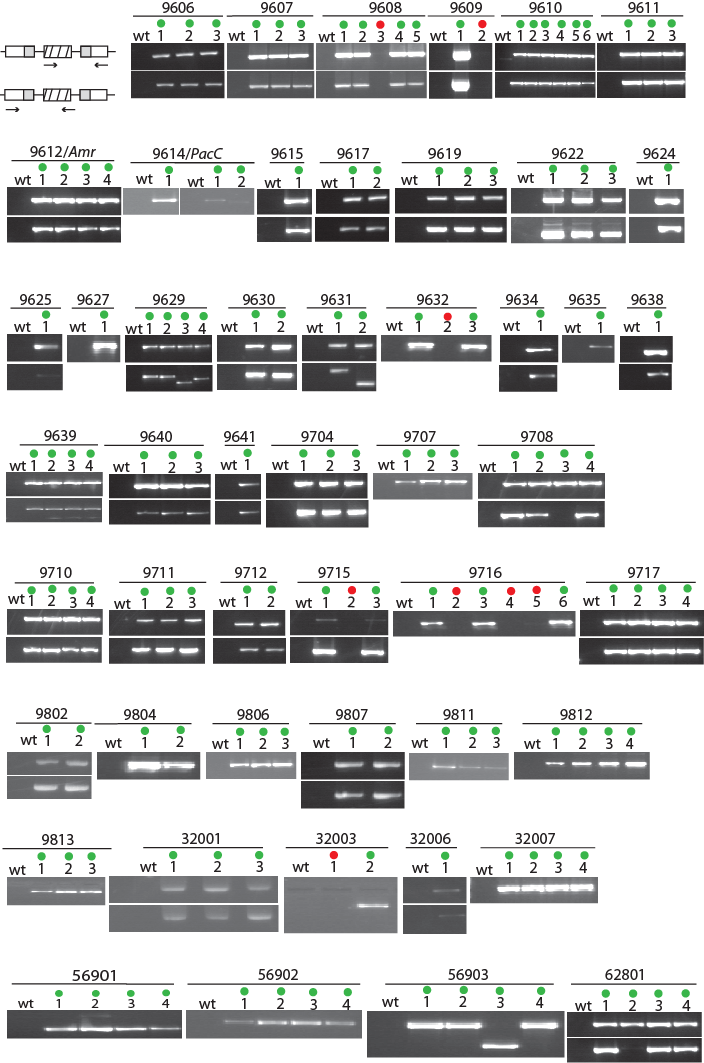


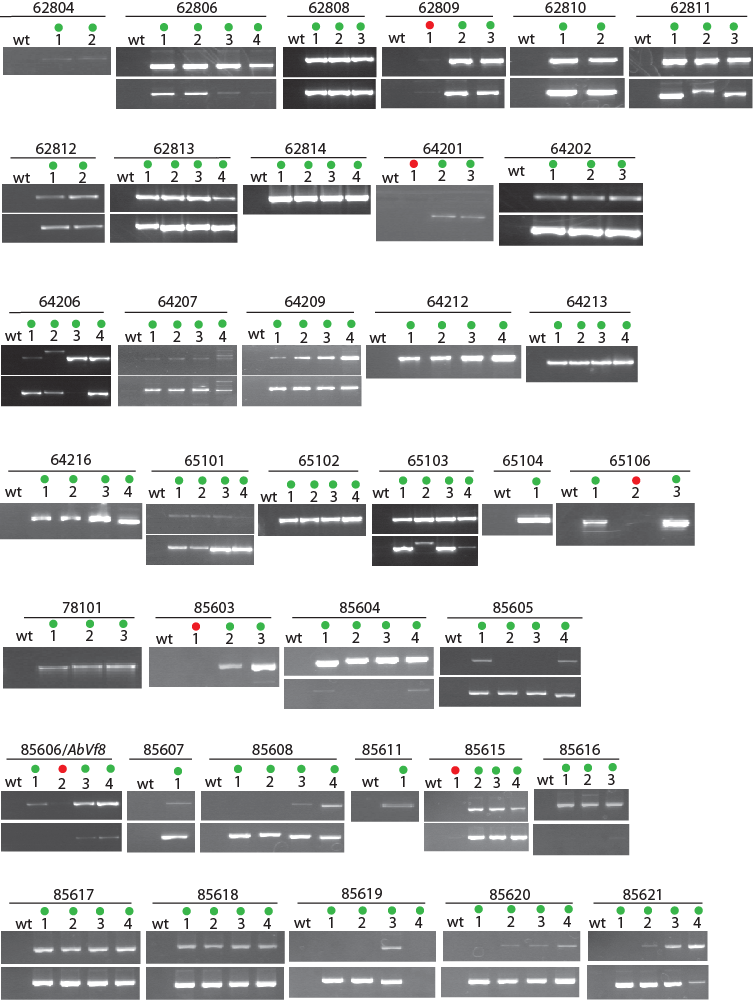


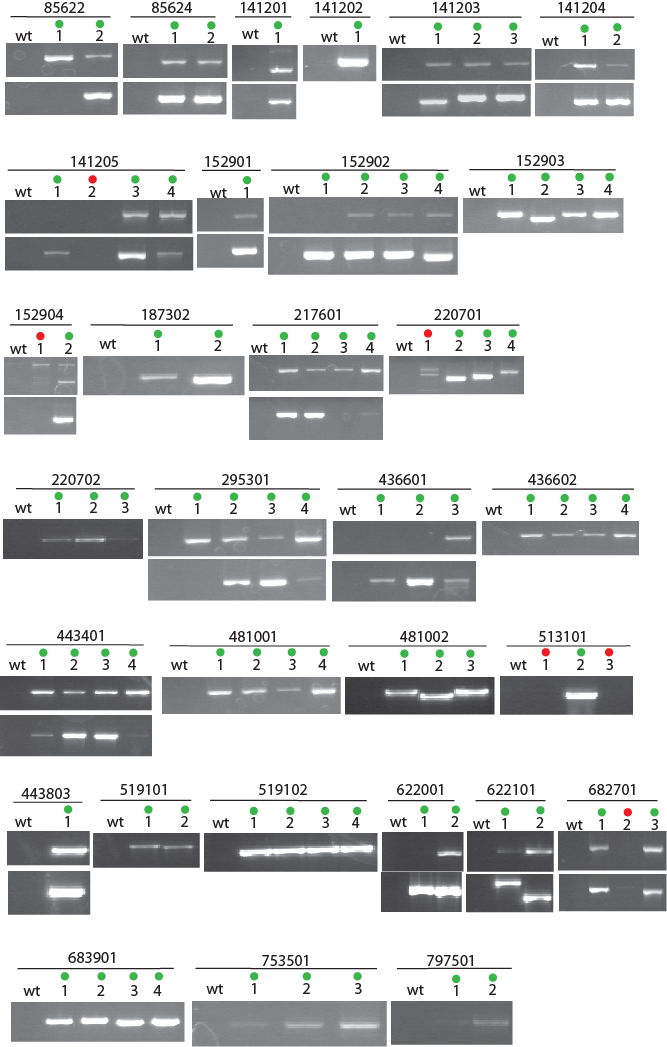

Supplement: Figure S1 — Verification of targeted gene disruption by polymerase chain reaction (PCR). PCR amplification was performed with two pairs of target gene specific and HygB resistance gene primers. Relative primer binding locations are marked below the schematic diagram of the disrupted target gene on the left side of the gel images at the top row of page one. The diagrams were drawn based on a hypothesis that the target gene was disrupted by a single homologous recombination with a circularized linear minimal element construct as described previously (Cho Y, Davis JW, Kim KH, Wang J, Sun QH, et al. (2006) A high throughput targeted gene disruption method for Alternaria brassicicola functional genomics using linear minimal element (LME) constructs. Mol Plant Microbe Interact 19: 7–15.). Hatched box = a selectable marker gene, white box = a target genomic locus, and gray box = partial target gene. Amplification was performed with Taq polymerase for PCR reactions (Invitrogen, Carlsbad, CA, U.S.A.). (DOC) [file ppat.1002974.s001.doc]
