# Supplementary material for: Transcription Factor Amr1 Induces Melanin Biosynthesis and Suppresses Virulence in Alternaria brassicicola
Source: PLoS Pathog. 2012 Oct 25;8(10):e1002974. doi: 10.1371/journal.ppat.1002974 (PMC3486909; doi:10.1371/journal.ppat.1002974)
Supplement: Figure S2 — Creation of Δabvf8 deletion mutants and pathogenicity assays. A. Schematic diagram of the wild-type locus of the AbVf8 gene, a replacement construct, and a mutant locus. The mutant locus depicts incorporation of the replacement construct into a wild-type locus by double homologous recombination resulting in gene replacement. B. Replacement of the AbVf8 coding region with the selectable marker, Hygromycin B (HygB) resistance cassette. Absence of a wild-type band in nine lanes on the AbVf8-probe blot (above) indicates loss of the targeted gene. On the HygB-probed blot (below), the expected 4.6 Kb band indicates a single-copy insertion of the Hyg B resistance cassette in 9 lanes. Asterisk (*) indicates lanes of mutants used in this study. Probe regions are marked by p-HygB and p-AbVf8. C. Lesions on Brassica oleracea 5 days after inoculation with ∼2,000 conidia of the wild type (wt), two Δabvf8 mutants, and an ectopic insertion mutant. Abbreviations: H = Hind III enzyme digestion site, wt = wild type Alternaria brassicicola, Ect = ectopic insertion mutant. (DOC) [file ppat.1002974.s002.doc]

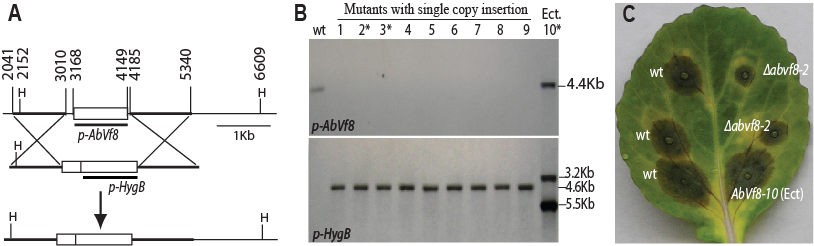


Figure S2. Creation of *∆abvf8* deletion mutants and pathogenicity assays. A. Schematic diagram of the wild-type locus of the *AbVf8* gene, a replacement construct, and a mutant locus. The mutant locus depicts incorporation of the replacement construct into a wild-type locus by double homologous recombination resulting in gene replacement. B. Replacement of the *AbVf8* coding region with the selectable marker, Hygromycin B (HygB) resistance cassette. Absence of a wild-type band in nine lanes on the *AbVf8*-probe blot (above) indicates loss of the targeted gene. On the HygB-probed blot (below), the expected 4.6 Kb band indicates a single-copy insertion of the Hyg B resistance cassette in 9 lanes. Asterisk (*) indicates lanes of mutants used in this study. Probe regions are marked by *p-HygB* and *p-AbVf8*. C. Lesions on *Brassica oleracea* 5 days after inoculation with ~2,000 conidia of the wild type (wt), two Δ*abvf8* mutants, and an ectopic insertion mutant. Abbreviations: H = *Hin*d III enzyme digestion site, wt = wild type *Alternaria brassicicola*, Ect = ectopic insertion mutant.
