# Supplementary material for: Transcription Factor Amr1 Induces Melanin Biosynthesis and Suppresses Virulence in Alternaria brassicicola
Source: PLoS Pathog. 2012 Oct 25;8(10):e1002974. doi: 10.1371/journal.ppat.1002974 (PMC3486909; doi:10.1371/journal.ppat.1002974)
Supplement: Figure S3 — Pigment secreted by Δamr1 mutants and its effect on pathogenesis and host plants. A. Mycelia of the Δamr1 mutant at the bottom of the flask (left) is melanin deficient compared to the dark-colored wild-type mycelia (right). Both were cultured in 1% glucose and 0.5% yeast extract broth (GYEB) for three days, in the dark, with continuous agitation at 100 rpm. B. No toxic effects from the pigment. The pigment in 10 ml of GYEB was extracted with 10 ml chloroform, twice. The chloroform was removed by evaporation and the pigment dissolved in 1 ml of sterile water. The concentrated pigment was injected into green cabbage leaves to evaluate its toxic effect. Both the pigment and negative control (water) produced similar small water-soaked areas that disappeared within 24 hours. Injected areas are marked with circles. C. No visible effect of the pigment on plant infection. Wild-type conidia were inoculated either in water (left) or in pigment (right). Lesion size was not significantly different between the two treatments. (DOC) [file ppat.1002974.s003.doc]

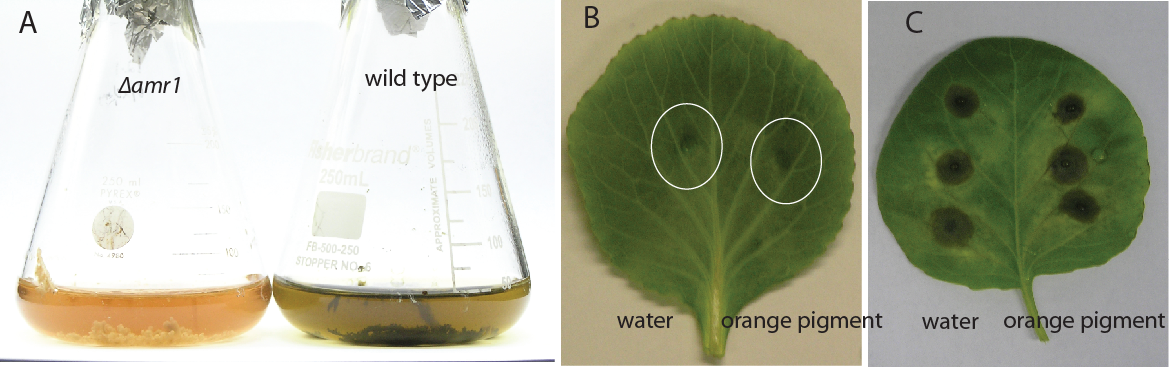


Figure S3. Pigment secreted by *∆amr1* mutants and its effect on pathogenesis and host plants. A. Mycelia of the *∆amr1* mutant at the bottom of the flask (left) is melanin deficient compared to the dark-colored wild-type mycelia (right). Both were cultured in 1% glucose and 0.5% yeast extract broth (GYEB) for three days, in the dark, with continuous agitation at 100 rpm. B. No toxic effects from the pigment. The pigment in 10 ml of GYEB was extracted with 10 ml chloroform, twice. The chloroform was removed by evaporation and the pigment dissolved in 1 ml of sterile water. The concentrated pigment was injected into green cabbage leaves to evaluate its toxic effect. Both the pigment and negative control (water) produced similar small water-soaked areas that disappeared within 24 hours. Injected areas are marked with circles. C. No visible effect of the pigment on plant infection. Wild-type conidia were inoculated either in water (left) or in pigment (right). Lesion size was not significantly different between the two treatments.
