# Supplementary material for: Transcription Factor Amr1 Induces Melanin Biosynthesis and Suppresses Virulence in Alternaria brassicicola
Source: PLoS Pathog. 2012 Oct 25;8(10):e1002974. doi: 10.1371/journal.ppat.1002974 (PMC3486909; doi:10.1371/journal.ppat.1002974)
Supplement: Figure S4 — Comparison of phenotypes between abpks7 mutants and wild-type Alternaria brassicicola . A. Stereoscopic view of an abpks7 mutant colony showing melanin-deficient aerial hyphae and conidia. B. Stereoscopic view of a wild-type colony showing melanized aerial hyphae and conidia. C. Hyaline conidia of an abpks7 mutant. Mutant conidia were mixed with a small number of dark, wild-type conidia to illustrate the color difference. D. Pathogenicity assay showing comparable lesions caused by three abpks7 mutants and wild-type A. brassicicola. Average lesion diameters caused by the mutants and wild type were not statistically different. (DOC) [file ppat.1002974.s004.doc]

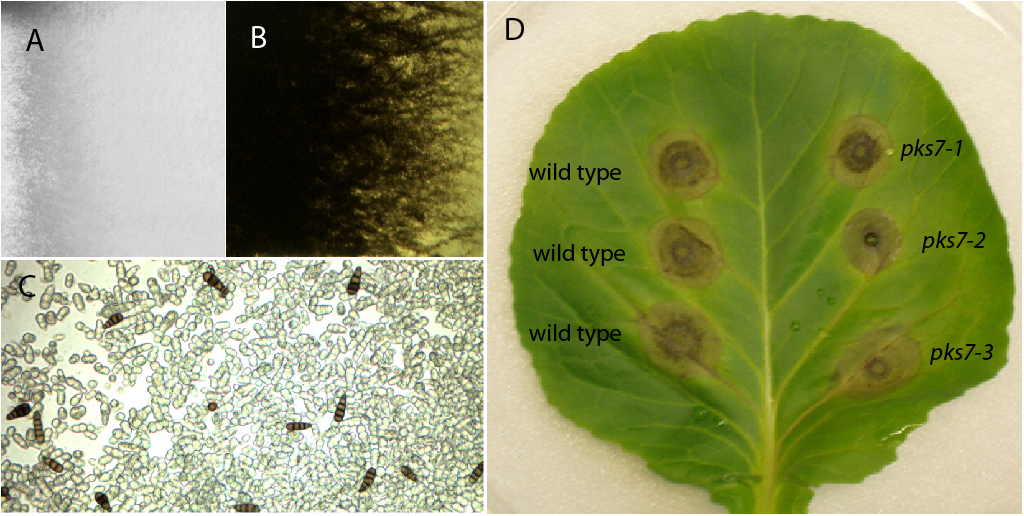


Figure S4. Comparison of phenotypes between *abpks7* mutants and wild-type *Alternaria brassicicola*. A. Stereoscopic view of an *abpks7* mutant colony showing melanin-deficient aerial hyphae and conidia. B. Stereoscopic view of a wild-type colony showing melanized aerial hyphae and conidia. C. Hyaline conidia of an *abpks7* mutant. Mutant conidia were mixed with a small number of dark, wild-type conidia to illustrate the color difference. D. Pathogenicity assay showing comparable lesions caused by three *abpks7* mutants and wild-type *A. brassicicola*. Average lesion diameters caused by the mutants and wild type were not statistically different.
