# Supplementary material for: Transcription Factor Amr1 Induces Melanin Biosynthesis and Suppresses Virulence in Alternaria brassicicola
Source: PLoS Pathog. 2012 Oct 25;8(10):e1002974. doi: 10.1371/journal.ppat.1002974 (PMC3486909; doi:10.1371/journal.ppat.1002974)
Supplement: Table S3 — Statistically over-represented functional annotation terms among 101 up-regulated genes in the Δamr1 mutant compared to the wild type. The 101 genes were selected based on the statistical analysis included in Cufflink with at least a two-fold-difference. (DOC) [file ppat.1002974.s007.doc]

Table S3. Statistically over-represented functional annotation terms among 101 up-regulated genes in the *∆amr1* mutant compared to the wild type. The 101 genes were selected based on the statistical analysis included in Cufflink with at least a two-fold-difference.

| Annotation term | Description | Up-regulated genes with the annotated term | Up-regulated genes excluding those with the annotated term | Other genes with the annotated term | | Other genes excluding those of the annotated term | representation | p-value (uncorrected) | p-value (corrected) |
| --- | --- | --- | --- | --- | --- | --- | --- | --- | --- |
| GO:0016798 | hydrolase activity, acting on glycosyl bonds | 15 | 87 | 228 | | 10,358 | Over-represented | 8.74E-09 | 1.25E-05 |
| GO:0005975 | carbohydrate metabolic process | 15 | 87 | 234 | | 10,352 | Over-represented | 1.22E-08 | 1.25E-05 |
| GO:0004553 | hydrolase activity, hydrolyzing O-glycosyl compounds | 15 | 87 | 224 | | 10,362 | Over-represented | 6.98E-09 | 1.25E-05 |
|  | | | | | | | | | |
| PF03443 | Glycoside hydrolase, family 61 | 6 | 96 | 6 | 10,580 | | Over-represented | 5.75E-10 | 1.06E-06 |
