# Supplementary material for: Transcription Factor Amr1 Induces Melanin Biosynthesis and Suppresses Virulence in Alternaria brassicicola
Source: PLoS Pathog. 2012 Oct 25;8(10):e1002974. doi: 10.1371/journal.ppat.1002974 (PMC3486909; doi:10.1371/journal.ppat.1002974)
Supplement: Table S4 — Disruption Primers (5′ to 3′ direction). (DOC) [file ppat.1002974.s008.doc]

Table S4. Disruption Primers (5’ to 3’ direction)

9606Dis1 CTGTGAGTAGGGGAGCGAAC

9606Dis2 atcagttaacgtcgacctcgTGGCTAGGAAGCTGTTGAGG

9606Dis3 CCTCAACAGCTTCCTAGCCACGAGGTCGACGTTAACTGAT

9607Dis1 TACGCATACACAGCCCTACC

9607Dis2 ATCAGTTAACGTCGACCTCGTGTCAACATGCTCCTGGAAT

9607Dis3 ATTCCAGGAGCATGTTGACACGAGGTCGACGTTAACTGAT

9608Dis1 ACTCTTGCGCACTGGAAGAT

9608Dis2 atcagttaacgtcgacctcgGGTGGCTGTGTTGAAAAGGT

9608Dis3 ACCTTTTCAACACAGCCACCCGAGGTCGACGTTAACTGAT

9609Dis1 CGGTAGGTCAGCGCATTTAT

9609Dis2 atcagttaacgtcgacctcgTTTGGTGGTTGTTCATGGTG

9609Dis3 CACCATGAACAACCACCAAACGAGGTCGACGTTAACTGAT

9610Dis1 GGCAACGCCAGATAGAGC

9610Dis2 atcagttaacgtcgacctcgAGTTGTCGTCGGTGCTGAAT

9610Dis3 ATTCAGCACCGACGACAACTCGAGGTCGACGTTAACTGAT

9611Dis1 CATCAACGAACACCCAGCTA

9611Dis2 atcagttaacgtcgacctcgCTCTGGTATCGCGAGCAATC

9611Dis3 GATTGCTCGCGATACCAGAGCGAGGTCGACGTTAACTGAT

9612Dis1 TGGACAGTCGTTCACCAGAG

9612Dis2 atcagttaacgtcgacctcgGACTCGCTGGATACCTTTCG

9612Dis3 CGAAAGGTATCCAGCGAGTCCGAGGTCGACGTTAACTGAT

9614Dis1 GCGAGGTTCAGAGTACAGTGC

9614Dis2 atcagttaacgtcgacctcgAGCGGAGCAATACGGAATC

9614Dis3 GATTCCGTATTGCTCCGCTCGAGGTCGACGTTAACTGAT

9615Dis1 GCTGGGCTTCCAAGGTTTAC

9615Dis2 atcagttaacgtcgacctcgTCGTGACATGGAGAATTGGA

9615Dis3 TCCAATTCTCCATGTCACGACGAGGTCGACGTTAACTGAT

9617Dis1 AAGAAGCGCAGGAGGTATCC

9617Dis2 atcagttaacgtcgacctcgGATCTTTGGCCTCTTGTTCG

9617Dis3 CGAACAAGAGGCCAAAGATCCGAGGTCGACGTTAACTGAT

9619Dis1 AATTCGTTGCTAACTCCTTCAA

9619Dis2 atcagttaacgtcgacctcgGTAAGGGGCAGGCTGTGAAC

9619Dis3 GTTCACAGCCTGCCCCTTACCGAGGTCGACGTTAACTGAT

9622Dis1 GCAGAGGCGCCTAGTGGT

9622Dis2 ATCAGTTAACGTCGACCTCGGACGTTCCCCCTGAACTTG

9622Dis3 CAAGTTCAGGGGGAACGTCCGAGGTCGACGTTAACTGAT

9624Dis1 TGGTGCAGACGATCTGAAAG

9624Dis2 ATCAGTTAACGTCGACCTCGACAAGCGGCCTTTGTACACT

9624Dis3 AGTGTACAAAGGCCGCTTGTCGAGGTCGACGTTAACTGAT

9625Dis1 ACTCTGCCTCGACAGGTTTC

9625Dis2 ATCAGTTAACGTCGACCTCGACGTGAGTTGGGGTTGTTGT

9625Dis3 ACAACAACCCCAACTCACGTCGAGGTCGACGTTAACTGAT

9627Dis1 GTCCGCCAGACGTCCAAC

9627Dis2 ATCAGTTAACGTCGACCTCGATCACCCGCTGTACAACCAT

9627Dis3 ATGGTTGTACAGCGGGTGATCGAGGTCGACGTTAACTGAT

9629Dis1 TGGTGCACACCGATGTTACT

9629Dis2 ATCAGTTAACGTCGACCTCGGCTACCCACTGTCCGTTCTC

9629Dis3 GAGAACGGACAGTGGGTAGCCGAGGTCGACGTTAACTGAT

9630Dis1 TCCAGTCGTGTGCGTTTTAG

9630Dis2 ATCAGTTAACGTCGACCTCGGAGGAGCAAGCACGTTGAAT

9630Dis3 ATTCAACGTGCTTGCTCCTCCGAGGTCGACGTTAACTGAT

9631Dis1 CTGCCTGGCTGCTTGTTATT

9631Dis2 ATCAGTTAACGTCGACCTCGGCGGATCATATGCTTTCTCG

9631Dis3 CGAGAAAGCATATGATCCGCCGAGGTCGACGTTAACTGAT

9632Dis1 ATCATGATGGGCACTTCACC

9632Dis2 ATCAGTTAACGTCGACCTCGTCTCCATGTCGTCCAGAGTG

9632Dis3 CACTCTGGACGACATGGAGACGAGGTCGACGTTAACTGAT

9634Dis1 AGAAGACCCATCAGCCAAGA

9634Dis2 ATCAGTTAACGTCGACCTCGACATTGGAAGGGACGTTCAC

9634Dis3 GTGAACGTCCCTTCCAATGTCGAGGTCGACGTTAACTGAT

9635Dis1 CATCATGTGGGGTATGTGGA

9635Dis2 ATCAGTTAACGTCGACCTCGCTCTCGAACAAGCGATAGGG

9635Dis3 CCCTATCGCTTGTTCGAGAGCGAGGTCGACGTTAACTGAT

9638Dis1 GTTGGCACACTACGGTTCG

9638Dis2 ATCAGTTAACGTCGACCTCGGGCGTATTGGCTTTGTTTTC

9638Dis3 GAAAACAAAGCCAATACGCCCGAGGTCGACGTTAACTGAT

9639Dis1 CCACCCATGCACAAATATCC

9639Dis2 ATCAGTTAACGTCGACCTCGCGTAAACGGTGGAAGCTGTT

9639Dis3 AACAGCTTCCACCGTTTACGCGAGGTCGACGTTAACTGAT

9640Dis1 AAGCCAATCTCCACCAACAC

9640Dis2 ATCAGTTAACGTCGACCTCGTCTGATGATGATGCGGAGAA

9640Dis3 TTCTCCGCATCATCATCAGACGAGGTCGACGTTAACTGAT

9641Dis1 CGACAAACTACGACTGAACACA

9641Dis2 ATCAGTTAACGTCGACCTCGTCCGATATAGTGAGAGCTTGC

9641Dis3 GCAAGCTCTCACTATATCGGACGAGGTCGACGTTAACTGAT

9642Dis1 TGCCAAGGAAGAGCCTAGAA

9642Dis2 ATCAGTTAACGTCGACCTCGGTCAAGTCGATTGGCCTGTT

9642Dis3 AACAGGCCAATCGACTTGACCGAGGTCGACGTTAACTGAT

9704DSF1 TGATTCGCACAACCAAGAAG

9704DSR2 atcagttaacgtcgacctcgTTCAGCGTTGTCAGCAAAAG

9704DSF3 CTTTTGCTGACAACGCTGAACGAGGTCGACGTTAACTGAT

9707DSF1 CAGCGTCTGCAACTGGTATG

9707DSR2 atcagttaacgtcgacctcgTGTCTTCGGAGTCGGAGTCT

9707DSF3 AGACTCCGACTCCGAAGACACGAGGTCGACGTTAACTGAT

9708DSF1 GTTTGAGGATGGCGTTGATG

9708DSR2 atcagttaacgtcgacctcgTAGCTTCCGTCATCCTCCAG

9708DSF3 CTGGAGGATGACGGAAGCTACGAGGTCGACGTTAACTGAT

9710DSF1 CCAGCACTCCTAGCCAGAAT

9710DSR2 atcagttaacgtcgacctcgTTCGGCACTGTCCTCTTCTT

9710DSF3 AAGAAGAGGACAGTGCCGAACGAGGTCGACGTTAACTGAT

9711DSF1 CACCCAGACGAGAGACCTGT

9711DSR2 atcagttaacgtcgacctcgAGTTTGGGTTGTGGCTTGAC

9711DSF3 GTCAAGCCACAACCCAAACTCGAGGTCGACGTTAACTGAT

9712DSF1 AACCTACCTACCGCGTCCTC

9712DSR2 atcagttaacgtcgacctcgTGTAGCGGATGAGACATGGA

9712DS3F TCCATGTCTCATCCGCTACACGAGGTCGACGTTAACTGAT

9715DSF1 TGGGCGCATATATTTCAACA

9715DSR2 atcagttaacgtcgacctcgAAATGGTAACCTCAGCATCG

9715DSF3 CGATGCTGAGGTTACCATTTCGAGGTCGACGTTAACTGAT

9716DSF1 GTGCCAGAGTTGCTGCATAG

9716DSR2 atcagttaacgtcgacctcgCGCGGGTGACCTATCATATT

9716DSF3 AATATGATAGGTCACCCGCGCGAGGTCGACGTTAACTGAT

9717DSF1 CGAAACCGTCCTATTTGACC

9717DSR2 atcagttaacgtcgacctcgGCATCGGGGTCTTTGATTAG

9717DSF3 CTAATCAAAGACCCCGATGcCGAGGTCGACGTTAACTGAT

9802DSF1 CCGACTGCTGTTATGTCGAA

9802DSR2 atcagttaacgtcgacctcgGTCTGACCGCACCCGTAG

9802DSF3 CTACGGGTGCGGTCAGACCGAGGTCGACGTTAACTGAT

9804DSF1 GTCGTTGCTCCTCTCTCCAC

9804DSR2 atcagttaacgtcgacctcgGATCCATGTCGAAGGCAATC

9804DSF3 GATTGCCTTCGACATGGATCCGAGGTCGACGTTAACTGAT

9806DSF1 CTGGGCCGACTAAATGTCTT

9806DSR2 atcagttaacgtcgacctcgCTCGTTGCGGGAGTAGTAGG

9806DSF3 CCTACTACTCCCGCAACGAGCGAGGTCGACGTTAACTGAT

9807DSF1 TCAGCAGACACCCTCTGTCA

9807DSR2 atcagttaacgtcgacctcgCCTTGACCTCGACTTTGACC

9807DSF3 GGTCAAAGTCGAGGTCAAGGCGAGGTCGACGTTAACTGAT

9811DSF1 ACAAGTCAACATGCGAGACG

9811DSR2 atcagttaacgtcgacctcgCGATGTGTCCTGTCTCTCCA

9811DSF3 TGGAGAGACAGGACACATCGCGAGGTCGACGTTAACTGAT

9812DSF1 ACACCGACTACCTCGAGCAT

9812DSF1 atcagttaacgtcgacctcgGCGTGTCTTGGGATCGTACT

9812DSF3 AGTACGATCCCAAGACACGCCGAGGTCGACGTTAACTGAT

9813DSF1 AGCAACTCGGCTGTTCGTAT

9813DSR2 atcagttaacgtcgacctcgTGAGCCGCAATATTCTCTCC

9813DSF3 GGAGAGAATATTGCGGCTCACGAGGTCGACGTTAACTGAT

32001DSF1 TGATCTTTCGTGTGCTTTCG

32001DSR2 atcagttaacgtcgacctcgGTTGTGGCTTGTGATGATCG

32001DSF3 CGATCATCACAAGCCACAACCGAGGTCGACGTTAACTGAT

32003DSF1 TACGACGATGACGACTTGGA

32003DSF1 atcagttaacgtcgacctcgTTCATCGACGAACTGCTTTG

32003DSF3 CAAAGCAGTTCGTCGATGAACGAGGTCGACGTTAACTGAT

32006DSF1 AGACGCCAACCATCAATTTC

32006DSR2 atcagttaacgtcgacctcgCGCGAGAGAAAAGTGCAAAG

32006DSF3 CTTTGCACTTTTCTCTCGCGCGAGGTCGACGTTAACTGAT

32007DSF1 CCCTATGGCACACTCTCCAG

32007DSR2 atcagttaacgtcgacctcgGTACAGACCACATGCGTTGC

32007DSF3 GCAACGCATGTGGTCTGTACCGAGGTCGACGTTAACTGAT

56901DSF1 TCAAGCTGCCTCTGAAGGAT

56901DSR2 atcagttaacgtcgacctcgCCCGAGTTGTAGTTGGCAGT

56901DSF3 ACTGCCAACTACAACTCGGGCGAGGTCGACGTTAACTGAT

56902DSF1 CGCTATGCACCCGCTATATT

56902DSR2 atcagttaacgtcgacctcgGTTCCCCGAATAGGCTTCTC

56902DSF3 GAGAAGCCTATTCGGGGAACCGAGGTCGACGTTAACTGAT

56903DSF1 TACGTCTGCGATGTCTGCTC

56903DSR2 atcagttaacgtcgacctcgGGGCATGTATCCTTGAACCT

56903DSF3 AGGTTCAAGGATACATGCCCCGAGGTCGACGTTAACTGAT

62801DSF1 GTGAAGTGAGGTCGGCATCT

62801DSR2 atcagttaacgtcgacctcgAGTCGACCATGACCCTCTTG

62801DSF3 CAAGAGGGTCATGGTCGACTCGAGGTCGACGTTAACTGAT

62802DSF1 CGCCAAACGATCAACCTATC

62802DSR2 atcagttaacgtcgacctcgGCATACCGTCTCAGAGCACA

62802DSF3 TGTGCTCTGAGACGGTATGCCGAGGTCGACGTTAACTGAT

62804DSF1 GTCTGTCACCACCCTGACTG

62804DSR2 atcagttaacgtcgacctcgGAGAGTATCGGCGTCCATGT

62804DSF3 ACATGGACGCCGATACTCTCCGAGGTCGACGTTAACTGAT

62806DSF1 GAGCAGTACAGGCGAGCTTT

62806DSR2 atcagttaacgtcgacctcgGCGGTTTGAGTGGAAACATT

62806DSF3 AATGTTTCCACTCAAACCGCCGAGGTCGACGTTAACTGAT

62808DSF1 CCGACCTCGACTCGACTTAC

62808DSR2 atcagttaacgtcgacctcgCACGAATCATCATCGTCTGG

62808DSF3 CCAGACGATGATGATTCGTGCGAGGTCGACGTTAACTGAT

62809DSF1 CCGGTAAGCAGACACCATCT

62809DSR2 atcagttaacgtcgacctcgTAGTTCTTGCCCTCCCAGAA

62809DSF3 TTCTGGGAGGGCAAGAACTACGAGGTCGACGTTAACTGAT

62810DSF1 CACAGGCACGAGAACGTCTA

62810DSR2 atcagttaacgtcgacctcgTCTCCTCTTTGTCCGCGTAT

62810DSF3 aTACGCGGACAAAGAGGAGACGAGGTCGACGTTAACTGAT

62811DSF1 CCAACTTGGCCCAGTACATC

62811DSR2 atcagttaacgtcgacctcgTGTCCTGCATTGATCCACAT

62811DSF3 ATGTGGATCAATGCAGGACACGAGGTCGACGTTAACTGAT

62812DSF1 CAACTCGTCATCCTCCGTCT

62812DSR2 atcagttaacgtcgacctcgTACCCTTTGGGACTTGATGC

62812DSF3 GCATCAAGTCCCAAAGGGTACGAGGTCGACGTTAACTGAT

62813DSF1 CAACGTTCAATCACCGAATG

62813DSR2 atcagttaacgtcgacctcgCATCATTGACAGCGATCTGG

62813DSF3 CCAGATCGCTGTCAATGATGCGAGGTCGACGTTAACTGAT

62814DSF1 GTCATCCGCCACAACTCTTT

62814DSR2 atcagttaacgtcgacctcgGAAGCTGGCTTCACCAAGAC

62814DSF3 GTCTTGGTGAAGCCAGCTTCCGAGGTCGACGTTAACTGAT

64201DSF1 TGTGATTGGCGCAATAGAAA

64201DSR2 atcagttaacgtcgacctcgTTCCACGCCTACACATCTGA

64201DSF3 TCAGATGTGTAGGCGTGGAACGAGGTCGACGTTAACTGAT

64202DsF1 GTCGTTCAGGTTTCCTCCAC

64202DsR2 atcagttaacgtcgacctcgGAGGGTTCGATTGGTTTTGA

64202DsF3 TCAAAACCAATCGAACCCTCCGAGGTCGACGTTAACTGAT

64206DsF1 ACCTCACCGGAGGAAAAAGT

64206DsR2 atcagttaacgtcgacctcgCGCTTGATGAGGAAACAGTG

64206DsF3 CACTGTTTCCTCATCAAGCGCGAGGTCGACGTTAACTGAT

64207DsF1 AATGTTGGTGAAGACGCACA

64207DsR2 atcagttaacgtcgacctcgGCTACACGACGGGTAAAGGA

64207DsF3 TCCTTTACCCGTCGTGTAGCCGAGGTCGACGTTAACTGAT

64209DsF1 GCTCGTCCAGTACCCTACCA

64209Ds2 atcagttaacgtcgacctcgGCTGAAAACCTCTGGAGTGC

64209DsF3 GCACTCCAGAGGTTTTCAGCCGAGGTCGACGTTAACTGAT

64212DsF1 CGCTTTGTTCGTGTTGATTG

64212DsR2 atcagttaacgtcgacctcgACATGAAAGCGGGTAGGTTG

64212DsF3 CAACCTACCCGCTTTCATGTCGAGGTCGACGTTAACTGAT

64213DsF1 CGCAAGGAGACAAACCTCAC

64213DsR2 atcagttaacgtcgacctcgCGTATCCGTTAGCCTCCAAA

64213DsF3 TTTGGAGGCTAACGGATACGCGAGGTCGACGTTAACTGAT

64216DsF1 CATCACCAGTTCCACGAATG

64216DsR2 atcagttaacgtcgacctcgCGACCGAAAAATTGGTGAAT

64216DsF3 ATTCACCAATTTTTCGGTCGCGAGGTCGACGTTAACTGAT

65101DsF1 CTGGACCTGAGCGATGATTT

65101DsR2 atcagttaacgtcgacctcgATGCCACACCTAGCGACTCT

65101DsF3 AGAGTCGCTAGGTGTGGCATCGAGGTCGACGTTAACTGAT

65102DsF1 TCGTTATCGTACCCGACCAT

65102DsR2 atcagttaacgtcgacctcgTCGTGGTGAAGGTGAGTGAC

65102DsF3 GTCACTCACCTTCACCACGACGAGGTCGACGTTAACTGAT

65103DsF1 CTTTCCCGTCCAACATCCT

65103DsR2 atcagttaacgtcgacctcgGGCCACCGCTGATGTATAGT

65103DsF3 ACTATACATCAGCGGTGGCCCGAGGTCGACGTTAACTGAT

65104DsF1 TCAAATGGTCGATACGGTCA

65104DsR2 atcagttaacgtcgacctcgCTGCAGATGCTGAAACAGGA

65104DsF3 TCCTGTTTCAGCATCTGCAGCGAGGTCGACGTTAACTGAT

65106DsF1 CGCTGATCAGAGGCAGAAAC

65106DsR2 atcagttaacgtcgacctcgGTGAGGTGTCCGATGCTGTA

65106DsF3 TACAGCATCGGACACCTCACCGAGGTCGACGTTAACTGAT

78101DsF1 TCGCAACTACCACATTCGTC

78101DsR2 atcagttaacgtcgacctcgCAAGCACTTTGACCCGTTTT

78101DsF3 AAAACGGGTCAAAGTGCTTGCGAGGTCGACGTTAACTGAT

85603DsF1 ATCTTCCGATTTGCACCAAC

85603DsR2 atcagttaacgtcgacctcgGGATGGAAGATGGGCTAGAA

85603DsF13 TTCTAGCCCATCTTCCATCCCGAGGTCGACGTTAACTGAT

85604DsF1 GCCTCCACAACGGTGTAAGT

85604DsR2 atcagttaacgtcgacctcgTGTGCGATCTGGTTCATCTC

85604DsF3 GAGATGAACCAGATCGCACACGAGGTCGACGTTAACTGAT

85605DsF1 CAGCTTATGTTGCCCTCTCC

85605DsR2 atcagttaacgtcgacctcgACTGATCGACTGCCTGCTTT

85605DsF3 AAAGCAGGCAGTCGATCAGTCGAGGTCGACGTTAACTGAT

85606DsF1 GTAGTCGCATCCCCAAGGTA

85606DsR2 atcagttaacgtcgacctcgTCCATAGCGAGAAACTCGTG

85606DsF3 CACGAGTTTCTCGCTATGGACGAGGTCGACGTTAACTGAT

85607DsF1 AGTATGCTGCGAAGGCTGTT

85607DsR2 atcagttaacgtcgacctcgGACGGAGCTGGGACATTAAA

85607DsF3 TTTAATGTCCCAGCTCCGTCCGAGGTCGACGTTAACTGAT

85608DsF1 ATGGCATTGAAGAGGTGTCC

85608DsR2 atcagttaacgtcgacctcgGCGCAGTCTTGTCATTTGAA

85608DsF3 TTCAAATGACAAGACTGCGCCGAGGTCGACGTTAACTGAT

85611DsF1 GAAAGCGTCGGTAATGGTGT

85611DsR2 atcagttaacgtcgacctcgTTCTGCGACAACGAGAATTG

85611DsF3 CAATTCTCGTTGTCGCAGAACGAGGTCGACGTTAACTGAT

85615DsF1 ACTGAACAAGCCGTCGAAGT

85615DsR2 atcagttaacgtcgacctcgTGGCTGGATTTCAAAGAAGG

85615DsF3 CCTTCTTTGAAATCCAGCCACGAGGTCGACGTTAACTGAT

85616DsF1 ATGACGACGAGGGAATCAAG

85616DsR2 atcagttaacgtcgacctcgTCGGTCTGCATACGACTGAG

85616DsF3 CTCAGTCGTATGCAGACCGACGAGGTCGACGTTAACTGAT

85617DsF1 CTCTGCTAGCCCTTGGTCAG

85617DsR2 atcagttaacgtcgacctcgGTTGCCCGAATCAGTTCCTA

85617DsF3 TAGGAACTGATTCGGGCAACCGAGGTCGACGTTAACTGAT

86518DsF1 GCCATGACTGCAGCACTAGA

86518DsR2 atcagttaacgtcgacctcgGAGGGTAGGTGAGGGGAAAG

86518DsF3 CTTTCCCCTCACCTACCCTCCGAGGTCGACGTTAACTGAT

85619DsF1 CGTTTCTTCACTGCACTCCA

85619DsR2 atcagttaacgtcgacctcgCACTGCTGTCATGGGATACG

85619DsF3 CGTATCCCATGACAGCAGTGCGAGGTCGACGTTAACTGAT

85620DsF1 CAACGGGAGGATAACACGAT

85620DsR2 atcagttaacgtcgacctcgGCATTGTTGCCCTGAATTTT

85620DsF3 AAAATTCAGGGCAACAATGCCGAGGTCGACGTTAACTGAT

85621DsF1 AGCGGAAATCAGTCCAAAGA

85621DsR2 atcagttaacgtcgacctcgCTCATCCCCGCTGAAAATTA

85621DsF3 TAATTTTCAGCGGGGATGAGCGAGGTCGACGTTAACTGAT

85622DsF1 CCGGTCTTCTTTGCAGTCTC

85622DsR2 atcagttaacgtcgacctcgCACGGCACCACTAGCACTAA

85622DsR2 TTAGTGCTAGTGGTGCCGTGCGAGGTCGACGTTAACTGAT

85624DsF1 GCTGGCAAGAAAGTCCACTC

85624DsR2 atcagttaacgtcgacctcgTGTAGTTTGCCAGGCAGTTG

85624DsF3 CAACTGCCTGGCAAACTACACGAGGTCGACGTTAACTGAT

141201DsF1 CCAACGACAACAACAAGTGC

141201DsR2 atcagttaacgtcgacctcgTAATCGCTTGGGCAGGTTAC

141201DsF3 GTAACCTGCCCAAGCGATTACGAGGTCGACGTTAACTGAT

141202DsF1 AGCGTACGTTACAGGACCTCA

141202DsR2 atcagttaacgtcgacctcgCGGTCCTCAGAGCTCGTACT

141202DsF3 AGTACGAGCTCTGAGGACCGCGAGGTCGACGTTAACTGAT

141203DsF1 TGCTCAGACTGCAAGAGGAA

141203DsR2 atcagttaacgtcgacctcgGAGCTTTGGGCTAGACGTTG

141203DsF3 CAACGTCTAGCCCAAAGCTCCGAGGTCGACGTTAACTGAT

141204DsF1 AAGACCGAGTCGCAAAACAT

141204DsR2 atcagttaacgtcgacctcgCGTTGGGGTACCTGGTAGAA

141204DsF3 TTCTACCAGGTACCCCAACGCGAGGTCGACGTTAACTGAT

141205DsF1 GCCTTTGTCTACTGCGCTTC

141205DsR2 atcagttaacgtcgacctcgGTTGTCGAGGTCCTTTGAGC

141205DsF3 GCTCAAAGGACCTCGACAACCGAGGTCGACGTTAACTGAT

152901DsF1 CCCAGTGGAGGAGGACATAA

152901DsR2 atcagttaacgtcgacctcgATTGATCCCTGGCTTCCTTT

152901DsF3 AAAGGAAGCCAGGGATCAATCGAGGTCGACGTTAACTGAT

152902DsF1 CGCACCATCATCATTTTCAC

152902DsR2 atcagttaacgtcgacctcgTGACACGCATGTCTTGCATA

152902DsF3 TATGCAAGACATGCGTGTCACGAGGTCGACGTTAACTGAT

152903DsF1 CCGGCTGTATACGTCCTGAT

152903DsR2 atcagttaacgtcgacctcgTGTCGAGGTGACGGTTGTAG

152903DsF3 CTACAACCGTCACCTCGACACGAGGTCGACGTTAACTGAT

152904DsF1 TCTGGTCGAAGCCAGATCTTA

152904DsR2 atcagttaacgtcgacctcgGAGAGCACCGATCCAAGTTC

152904DsF3 GAACTTGGATCGGTGCTCTCCGAGGTCGACGTTAACTGAT

187302DsF1 CCAGCAAGCTCCAGGACTAC

187302DsR2 atcagttaacgtcgacctcgTTGGCGTCTTCCTCTTCAAT

187302DsF3 ATTGAAGAGGAAGACGCCAACGAGGTCGACGTTAACTGAT

217601DsF1 CAACTGTCCTGCGAGCACTA

217601DsR2 atcagttaacgtcgacctcgGGATTTCAGCCAAACAGAGC

217601DsF3 GCTCTGTTTGGCTGAAATCCCGAGGTCGACGTTAACTGAT

220701DsF1 TACCAGCAGCATATCGCAAG

220701DsR2 atcagttaacgtcgacctcgGAAGCCTACCAGGCACTCAG

220701DsF3 CTGAGTGCCTGGTAGGCTTCCGAGGTCGACGTTAACTGAT

220702DsF1 TTGCACGAGCAGGAACTATG

220702DsR2 atcagttaacgtcgacctcgAGTGAACAGGCCGTATCCAG

220702DsF3 CTGGATACGGCCTGTTCACTCGAGGTCGACGTTAACTGAT

295301DsF1 TCTGAGGTCTGCAGGGTCTT

295301DsR2 atcagttaacgtcgacctcgTATGTCCACTCGAGCTGTCG

295301DsF3 CGACAGCTCGAGTGGACATACGAGGTCGACGTTAACTGAT

436601DsF1 GCCCTCTTCCATGAGCAGTA

436601DsR2 atcagttaacgtcgacctcgGACATTGCCACCAAGAGTCA

436601DsF3 TGACTCTTGGTGGCAATGTCCGAGGTCGACGTTAACTGAT

436602DsF1 CAAGACGCAGTCAAAGCAAG

436602DsR2 atcagttaacgtcgacctcgGACTGCGCTGATTTCTCCTC

436602DsF3 GAGGAGAAATCAGCGCAGTCCGAGGTCGACGTTAACTGAT

443401DsF1 GCAAAGTTCACCTCCTCGTC

443401DsR2 atcagttaacgtcgacctcgGTTGGGGTGATAATGCAAGG

443401DsF3 CCTTGCATTATCACCCCAACCGAGGTCGACGTTAACTGAT

443803DsF1 GTCGAAGTGCTCCCAAACTC

443803DsR2 atcagttaacgtcgacctcgTTGATCTGCCGCTGATACTG

443803DsF3 CAGTATCAGCGGCAGATCAACGAGGTCGACGTTAACTGAT

481001DsF1 TCATTGGAGAAATGGGAACC

481001DsR2 atcagttaacgtcgacctcgTAGCGGAGTGTCCGTCTTCT

481001DsF3 AGAAGACGGACACTCCGCTACGAGGTCGACGTTAACTGAT

481002DsF1 AAATCTTCCAGACGCAAACG

481002DsR2 atcagttaacgtcgacctcgATCTGTTCGGGGTCTACACG

481002DsF3 CGTGTAGACCCCGAACAGATCGAGGTCGACGTTAACTGAT

513101DsF1 CTGTGGTCCTTGACCAGGTT

513101DsR2 atcagttaacgtcgacctcgTATACCGTCTTGCGGTCCAT

513101DsF3 ATGGACCGCAAGACGGTATACGAGGTCGACGTTAACTGAT

519101DsF1 TGCCTATCCAGAGGTTGTCC

519101DsR2 atcagttaacgtcgacctcgGAGCTGAAGTTGAGCGCATA

519101DsF3 TATGCGCTCAACTTCAGCTCCGAGGTCGACGTTAACTGAT

519102DsF1 ACCCCACCTGTTCACATGAT

519102DsR2 atcagttaacgtcgacctcgGACAGCCTGTTCTCCAGCTC

519102DsF3 GAGCTGGAGAACAGGCTGTCCGAGGTCGACGTTAACTGAT

622001DsF1 CAGCCAAAATACTGGTGCAA

622001DsR2 atcagttaacgtcgacctcgCTCCCATTGTCGTTTCCTGT

622001DsF3 ACAGGAAACGACAATGGGAGCGAGGTCGACGTTAACTGAT

622101DsF1 CTGTCGAAGCTTCTGCCTCT

622101DsR2 atcagttaacgtcgacctcgCTTTGCCTTTGGTTTCGTGT

622101DsF3 ACACGAAACCAAAGGCAAAGCGAGGTCGACGTTAACTGAT

682701DsF1 GTGGCGACCATCTCTTGAGT

682701DsR2 atcagttaacgtcgacctcgTGGACTACCGTCCTTTGCTC

682701DsF3 GAGCAAAGGACGGTAGTCCACGAGGTCGACGTTAACTGAT

683901DsF1 CAAGAGGAAGTGGGCTGAAC

683901DsR2 atcagttaacgtcgacctcgGACGCGTCTTGCAGTAGTTG

683901DsF3 CAACTACTGCAAGACGCGTCCGAGGTCGACGTTAACTGAT

753501DsF1 CAATCTGCAAAACGCTACCA

753501DsR2 atcagttaacgtcgacctcgACGCATCTTCACCAGTCTCC

753501DsF3 GGAGACTGGTGAAGATGCGTCGAGGTCGACGTTAACTGAT

797501DsF1 GTATGCACGATGGTGTCAGC

797501DsR2 atcagttaacgtcgacctcgCTGGAGCCAATGTACGGATT

797501DsF3 AATCCGTACATTGGCTCCAGCGAGGTCGACGTTAACTGAT
