# Supplementary material for: Transcription Factor Amr1 Induces Melanin Biosynthesis and Suppresses Virulence in Alternaria brassicicola
Source: PLoS Pathog. 2012 Oct 25;8(10):e1002974. doi: 10.1371/journal.ppat.1002974 (PMC3486909; doi:10.1371/journal.ppat.1002974)
Supplement: Table S5 — PCR verification primers (5′ to 3′ direction). (DOC) [file ppat.1002974.s009.doc]

Table S5. PCR verification primers(5’ to 3’ direction)

HygIn1343R ATTTGTGTACGCCCGACAGT

HygIn84F CTTGGCTGGAGCTAGTGGAG

VHyg231R GCACCAAGCAGCAGATGATA

VHyg962F ATTTCGGCTCCAACAATGTC

9606VF1 GGCAATTGACCTTTTGCTGT

9606VR2 ATTCGTACAAAGCGCCAATC

9607VF1 GCAACAAAGGCTGGGTACAT

9607VR2 CTTAACAACGGCACCATCCT

9608VF1 ACAACCCCAATGCAACGTAT

9608VR2 CCATGGCACAATCTCAACAC

9609VF1 CCGGCTACTAGACGCTTCAC

9609VR2 GTCGGATACATACGCGACCT

9610VF1 TTAGAGGCGAAACTGCTGGT

9610VR2 CCTTCATAAAGGCAGGTCCA

9611VF1 ACAGCGAGCAAAGACATCCT

6911VR2 CTGGGCTGCATGTACTGAGA

9612VF1 CGACTCCCCTCTCGTGATAA

9612VR2 CAGGCGGAAGAAGAACAAAG

9614VF1 GTAGTATAACCCGCCGACGA

9614VR2 GTTCTCAACCCACGATTGCT

9615VF1 TCGTCTCGCAATTACTCGTG

9615VR2 CATGTAGGCCTGATCCTGGT

9617VF1 TAGCAAGCAAGGGCGATACT

9617VR2 GGTTAGGGTGCACGACTGTT

9619VR2 CCGGGCGTTTTGTAAGAGTA

9622VF1 GAACTTCATGCTTGGGGAAA

9622VR2 GAGGTCTTCCGTAGGTGCTG

9624VF1 GCCTTCGAAGTTGTCTTTGG

9624VR2 GTTGGTGGCAAACGTCTTCT

9625VF1 GACCCGCATTTCTTGATACG

9625VR2 TGAATGACGGAATGCAAAAA

9627VF1 TCGATCCCAAGACTCCAATC

9627VR2 GATGTCGTTCTCCACGGATT

9629VF1 GGCGCGATTATTCTGATTGT

9629VR2 ATGTTGCTGGCCCACTAAAC

9630VF1 AGAGTAAAGTTGCCGCTGGA

9630VR2 ACGTACGTCAGGCGAGAACT

9631VF1 TCGCGATTGCCTAGCTAAAT

9631VR2 GTTGTGCTTCGTCATGTGCT

9632VF1 CATGTGCAGCACTCCAATCT

9632VR2 GACATTTGCGTTTTGGAGGT

9634VF1 AGGCGATAGATACCGTGTGG

9634VR2 CGTTTATGGTGGCCTCTGTT

9635VF1 GCTCCGGGTTCTAAATCTCC

9635VR2 CAGGTTCTGGCTCTCCTGAC

9638VF1 GCATTTCGAAACAACCCAGT

9638VR2 TGATGCTTCCGCTTGTACTG

9639VR2 GCTGTGCTGAAAGCAACAAA

9640VF1 GCAGAACATCCCAAAGGTGT

9640VR2 CAACAGCACGTCCATGAATC

9641VF1 GGGCTTGTATATGGGCTGAA

9641VR2 ACACGATTTTCCCGTACTGC

9704VF1 ACACCCTCCACTGTCTTTGG

9704VR1 GGACGTCAGCAACAGACTCA

9707VF1 GCCCTTCCACAATTAGTGCT

9707VR1 CCGGAACCTTCTTCAAATCA

9708VF1 AGCGTCCGCTACTTAAACCA

9708VR1 CTTTTGGTGTCTTCGGTGGT

9710VF1 CCATGACGATGTGCCAATAG

9710VR1 GCGGTAGACGAGGTGAAGAG

9711VF1 AAGGCACAAAGAATGGATGG

9711VR1 GATGTAAGGTCGGCAGGTGT

9712VF1 AGAACGCCGTCATCGTTATC

9712VR1 TGTTTGGTATTGGCGTGAAA

9715VF1 GCACAACCATAGTTGCATGG

9715VR1 AATCCTTTTGCGACCACAAC

9716VF1 TCCGACCGTCTCTATTCTCG

9716VR1 AAAGTTGAAGCCACCCATTG

9717VF1 CAGAACACACTCAGCCGAAA

9717VR1 TTTCCGCAGAGATCCCATAC

9802VF1 GCATTGCTGATTGTCTGCAT

9802VR1 AGAAGCATCAACCCAACCAC

9804VF1 GTCGAACAACGCTCAAGACA

9804VR1 AGCGACCTGTTTCATGCTCT

9806VF1 TAAATCTTCAGCCCCTGTGG

9806VR1 GCTAGAAGAACCGCCTTCCT

9807VF1 GCCGAAGGTGACAGAAGAAG

9807VR1 TGACACAGATGGTTGGTCGT

9811VF1 CACTGCCAATCTGCAACACT

9811VR1 GTAGGGTTCTCGGACCATGA

9812VF1 CCACTAATTCGCGATCCACT

9812VR1 TGGCTTGCAAATGTAGCTTG

9813VF1 CCTGCGCAAATGATAATTCA

9813VR1 CGCGGAGTTACCTTTCTGAG

32001VF1 CGACTGGGGTTTGATATGCT

32001VR1 ACAACGAAGGTGGGTTTCTG

32003VF1 ACATTTCCGACACGAACCAT

32003VR1 TGAAGCTCCTCGGAAGGTAA

32006VF1 CACAGCTTCCACACTGGCTA

32006VR1 TGACGTCGGTCTTGAGTGAG

32007VF1 AAGTCCGGACATCTGTTTGC

32007VR1 GTGGCTCGGGTGTGTAATCT

56901Vf1 GGCCAGACGAAGAAATTCAA

56901VR1 CTCTTCAGCCTTGACCTTGG

56902Vf1 CAACACCCTCTACCGTGTCC

56902Vr1 GCTCAATTAGCGGCAGATTC

56903Vf1 CACCACGTCCACTTGACATC

56903Vr1 GATCCGCTTTCGACTCTTTG

62801Vf1 GAACGATGATGCAAGAAGCA

62801Vr1 ACGGCCTTCTTGAGCTTGTA

62802Vf1 GCTTCAGGCTTTGGAAGATG

62802Vr1 TTGAAGTATGCGCGTTTCTG

62804Vf1 CACAATGAGCAGCATCGACT

62804VR1 CTGCTTGCCGATTTTGGTAT

62806Vf1 CATCTGTCTGAGACGCCAAA

62806VR1 TTTGCCTTTGGCTCTTCTGT

62808Vf1 GCTCAGACGACTGTGAACGA

62808VR1 TACAGGCGTATTTGCTGCTG

62809Vf1 CTCGTTCTGGCAATCCTCTC

62809VR1 CAGGGTGTCACGGAGTTTTT

62810Vf1 CGTCATTCATACCACGCAAC

62810VR1 CTGCTCAAGCTCAGTGATCG

62811VR1 TTCGAGGCTAGCTTCTGCTC

62812Vf1 CGTCGCTCTCTAAAGCTCGT

62812VR1 CCTATTGTTGCGGTCCTTGT

62813VF1 GTCCACCAACGAGAAGAAGC

62813VR1 GACTCAGGCGAGGTATCAGC

62814Vf1 GCCCCAATCAAACGTCTCTA

62814VR1 ATACCCGCGGCTTTCTTATT

64201Vf1 GGCTGGAAATCAGAAGACCA

64201VR1 TGGAAGCCTGTGTCTGTGTC

64202Vf1 CATCTTCTGCAACAGCCTTG

64202VR1 GGCTGCAAGTCCTTCTTGTC

64206Vf1 AGATGCGGGCACTTATCAAC

64206VR1 ATCCATGCCCTCATCAGAAG

64207Vf1 ATTAGTGCCCAGTCGAGGTG

64207VR1 GGCAGTCGTCTCCGTTAGAG

64209Vf1 ATACAGCTGCACCTGCACAC

64209VR1 CGATGAGACGACAGTGGCTA

64212Vf1 GCATTCCACATGCATCAATC

64212VR1 TAGACGATCCAGTCGTGCTG

64213Vf1 ACTTGTAATCACCCCCGACA

64213VR1 CGTCTGAGACAGCCATACGA

64216Vf1 CAGCTTCCGATTGACACAGA

64216VR1 AGATAGCGAACGCTTTTCCA

65101Vf1 TGAATCCACTTTTTGCCACA

65101VR1 CCTTCGATCCGAGACTTCTG

65102Vf1 GGCAGTTTCTCCAACCAAAA

65102VR1 TAGGCAAAGATGACGTGCTG

65103Vf1 CGGCTAGCCAACTCAACTTC

65103VR1 GCAACCCGATGAGAAGATGT

65104Vf1 GACTCGTTTTCGCCTCAGTC

65104VR1 GCTAGAGGCGCTTCAAGAGA

65106Vf1 GGTCGAACGCCAAGATGTAT

65106VR1 CTGTCCTTAATCCGCCTGAG

78101Vf1 GATTCACGTTCCAGCCAGAT

78101VR1 GGCGGACATGTAGGCTATGT

85602Vf1 GTCCATGGAAGCTCCAACAT

85602VR1 GCTGTACCATCCGAAGGTGT

85603Vf1 GGAATAGCGCTGACTTGAGG

85603VR1 CTCAACTTCCTGCCTTCGTC

85604Vf1 TCCTGGTAGACCGCGTAAGT

85604VR1 CCACTGCTGCGTATTCAAGA

85605Vf1 ACAGTCCCAAACCTCACTGG

85605VR1 CCCGATCTTCGATACCAAAA

85606Vf1 GCGCTTCCAAATATGTTTCC

85606VR1 AATGCGATTCAGCTTCGAGT

85607Vf1 CAAAGCAACCACTTCAGCAA

85607VR1 AGTACTGGGGTCGAATGCAC

85608Vf1 GCATTTGCGCCTTTAACAAT

85608VR1 CTGTTTCCCCATGAACTCGT

85611Vf1 AGTCGCGAAACTCACGTTTT

85611VR1 GGGTACGGATGCTCACTTGT

85615Vf1 TCTCTCACCCACCTCGACTT

85615VR1 GCTTGAGCCAGACTTTGGAC

85616Vf1 GACGGACAAGAAGGGTTTTG

85616VR1 CGACGTGATGCTCGCTATAA

85617Vf1 TCGCCGTTTTCGTTTTCTAT

85617VR1 GCACGACAGCGTCTTTATGA

85618Vf1 GATTGCTACGCCATCGATTT

85618VR1 GTAGCAGTCATCTGCGGTCA

85619Vf1 GGCATACCAACAACTCGTCA

85619VR1 GGTGTTGTACGCATTGTTGC

85620Vf1 GGGCTCAAATCCGTTTACAA

85620VR1 CTCTTGGAAGAAGCGTTTGG

85621Vf1 CCACACATCTTCGCAATCAC

85621VR1 GGTTTGAGCGCTTTCATCTC

85622Vf1 CAACATGGTTCCAACACAGC

85622VR1 GCGCGACACAAACAAGAATA

85624Vf1 TCTCACATGGGAACCTGACA

85624VR1 GTCGAGTAACGCGCACTGTA

141201Vf1 GCATAGAGGATGGGTCGTGT

141201VR1 TGTCGTTGCTTCGTGTCTTC

141202Vf1 TCCTCCTTCCAGACCAGATG

141202VR1 GAGTTGGACGAAGAGGCTTG

141203Vf1 CTCGCACCATGCTACTGAAA

141203VR1 CGGTGACTTTGCACTTGAGA

141204Vf1 GAAGGCGTGCTATTGAGGAG

141204VR1 CCCTTTTGTGTGCCAAACTT

141205Vf1 TGTACATGTAGGCGGCAAGA

141205VR1 CGCGTTTGAGGACTTTCTTC

152901Vf1 TTGACCTCGTATTCGCAGTG

152901VR1 GTCCGTAGAACACCGCAAAT

152902Vf1 CTGGTCTGCTCTCACCATGA

152902VR1 CAACGGTGAAAGGGCATAGT

152903Vf1 ACTTTGGTGTACGGCGAAAC

152903VR1 AAGTGAGGGAAGGTGGTGTG

152904Vf1 CTTCCTCATGGTAGCGTGGT

152904VR1 CCACTCAAACTCACCCCTGT

187302Vf1 GAACTCGGAGCTCAGACCAA

187302Vr1 ATTCTCGAGCCAGTCCTTGA

217601Vf1 CAACCTCGTTTGCCTCATCT

217601VR1 GCGTTGACTCTCGTTGATGA

220701Vf1 CTTCCTTCCAGACGGAGTTG

220701VR1 GCTTTGCGTTTCTTGGAGTC

220702Vf1 ACCGCTACCAACAAACGAAC

220702VR1 ATGCCATTGAAGAAGCCATC

295301Vf1 CTTGATCGTCTTCAGCACCA

295301VR1 ATTGATTCTCAAGGGCATCG

436601Vf1 CCTTGTAATGTGTGGCGATG

436601VR1 GGCCTGTTGTCTGTCCAAAT

436602Vf1 CTTGGTAATGCCCAGGGTTA

436602VR1 GGACGCCACTGATGGTACTT

443401Vf1 TCCGCCCCATTAATTAGAAA

443401VR1 CCGAAAGTAGGGGAGGAAAG

443803Vf1 CCAAGGCCATGAATGAAGAT

443803VR1 TCATCTGCATTTGCGTCTTC

481001Vf1 TCTGCTGATTCCCAACCTCT

481001VR1 CAAGCTGTCCTCCTCCTGAC

481002Vf1 ATCATCCCGTTTGTCGAATC

481002VR1 GAACGTTACAATGCCGACCT

513101Vf1 AGAACTCAAATCGCGAGGAA

513101VR1 GAGGCTTTGGTGTGGTTGTT

519101Vf1 CGTTCCGGACACTCCATACT

519101VR1 TTCTGGGAAAGCTGTCGAGT

519102Vf1 GCGCTTAACTGAGGACAAGG

519102VR1 GTCTGGACACCAACACAACG

622001Vf1 TGGCTCCATGAGTGTCAGAG

622001VR1 GTACGCCCTTGTTCAACGAT

622101Vf1 GTGATGAATCACAGCCAACG

622101VR1 TTTGGCTTTTGCGAGTTTCT

682701Vf1 CGTACGAGCTCCCTGCTATC

682701VR1 AGGATATCAAAGCCGTGGTG

683901Vf1 GCGCGTGGAGAGTATCTAGG

683901VR1 GATGGGGATCGATAGCTTGA

753501Vf1 CCCTCCCAGCATACCATCTA

753501VR1 ACATCATTTGTGACGGACGA

797501Vf1 ATTTGTCAGAGGCGAGGATG

797501VR1 GGGGGAAGAGATGGTGGTAT
