# Supplementary material for: Transcription Factor Amr1 Induces Melanin Biosynthesis and Suppresses Virulence in Alternaria brassicicola
Source: PLoS Pathog. 2012 Oct 25;8(10):e1002974. doi: 10.1371/journal.ppat.1002974 (PMC3486909; doi:10.1371/journal.ppat.1002974)
Supplement: Table S6 — List of primers used for qRT-PCR and transformation constructs. (DOC) [file ppat.1002974.s010.doc]

Table S6A. List of primers used for qRT-PCR

| Gene name | GenBank number | | | Primer Name | Primer sequence (5’ to 3’) |
| --- | --- | --- | --- | --- | --- |
|  | *Alternaria brassicicola* Genome sequence | | Homolog |  |  |
| Actin | ACIW01001686.1  Ab05847.1 | [XP_001257681.1](http://www.ncbi.nlm.nih.gov/protein/119467750?report=genbank&log$=prottop&blast_rank=1&RID=PDJ09ARG01N) (E = 0.0) *Neosartorya fischeri*  [XP_664146.1](http://www.ncbi.nlm.nih.gov/protein/67540744?report=genbank&log$=prottop&blast_rank=3&RID=PDJ09ARG01N) (E = 0.0) *Aspergillus nidulans* | | ActinRTF | GGCAACATTGTCATGTCTGG |
|  |  |  |  | ActinRTR | GAGCGAAGCAAGAATGGAAC |
| Chymotrypsin | ACIW01000483.1  Ab01734.1 | [XP_001792474.1](http://www.ncbi.nlm.nih.gov/protein/169598102?report=genbank&log$=prottop&blast_rank=1&RID=PDK3BY3C01S) (E = 3e-159) *Phaeosphaeria nodorum*  [CAB44651.1](http://www.ncbi.nlm.nih.gov/protein/5042248?report=genbank&log$=prottop&blast_rank=3&RID=PDK3BY3C01S) (E = 7e-125) *Metarhizium anisopliae* | | ChymoRTF | CGGTACCACTGGAAACACT |
|  |  |  |  | ChymoRTR | TGGGTGAGACCAGTAACACG |
| Glycosyl hydrolase | ACIW01002530.1  Ab08726.1 | [XP_001937210.1](http://www.ncbi.nlm.nih.gov/protein/189201748?report=genbank&log$=prottop&blast_rank=1&RID=PDM7T5U701S) (E = 0.0) ) *Pyrenophora tritici-repentis*  [CBX97708.1](http://www.ncbi.nlm.nih.gov/protein/312217761?report=genbank&log$=prottop&blast_rank=3&RID=PDM7T5U701S) (E = 0.0) *Leptosphaeria maculans* | | GlycosylRTF | GTGGCATGCAATATGGACAA |
|  |  |  |  | GlycosylRTR | GCGGTAGAAATTTGCCTTGA |
| Cellobiohydrolase, CBH7 | [ACIW01001797.1](http://www.ncbi.nlm.nih.gov/nucleotide/224805926?report=genbank&log$=nucltop&blast_rank=1&RID=PDSGCBP8016)  Ab06252.1 | [XP_001933777.1](http://www.ncbi.nlm.nih.gov/protein/189194878?report=genbank&log$=prottop&blast_rank=2&RID=PDRE6N2Z01N) (E = 0.0) *Pyrenophora tritici-repentis*  AAM76664 (E = 0.0) *Cochliobolus heterostrophus* | | CBH7RTF | CGTCCGAGGTTTACACCCTA |
|  |  |  |  | CBH7RTR | ATGGTACCCATCAGCTTTGC |
| Lipase | ACIW01000050.1  Ab00164.1 | [XP_001940336.1](http://www.ncbi.nlm.nih.gov/protein/189208005?report=genbank&log$=prottop&blast_rank=1&RID=PDNKC6TF01N) (E = 0.0) *Pyrenophora tritici-repentis*  [CBX98811.1](http://www.ncbi.nlm.nih.gov/protein/312218866?report=genbank&log$=prottop&blast_rank=3&RID=PDNKC6TF01N) (E= 0.0) ) *Leptosphaeria maculans* | | LipaseRTF | CATTCTGGGGACGTTCCAT |
|  |  |  |  | LipaseRTR | CTGTTCGCTCCGAAGTTCAT |
| Alternaria melanin regulation | ACIW01000615.1  Ab02276.1 | [BAF95183.1](http://www.ncbi.nlm.nih.gov/protein/161958693?report=genbank&log$=prottop&blast_rank=1&RID=PDHA49DK014) (E = 0.0) *Bipolaris oryzae*  XP_001933651 (E = 0.0) *Pyrenophora tritici-repentis* | | Amr1RTF1 | CACCCAAAGCTTCCTCCATA |
|  |  |  |  | Amr1RTR1 | CAGGCGGAAGAAGAACAAAG |
| Alternaria melanin regulation | Same as above | Same as above | | Amr1RTF2 | TGTATCGGGCAGGGTTAGTC |
|  |  |  |  | Amr1RTR2 | AGCATTAGATGGCTGCTGGT |
| T3HN reductase (*Brn1*) | [ACIW01000615.1](http://www.ncbi.nlm.nih.gov/nucleotide/224807108?report=genbank&log$=nucltop&blast_rank=1&RID=PC7USFXM014)  Ab02276.1 | [BAA36503.1](http://www.ncbi.nlm.nih.gov/protein/4115722?report=genbank&log$=prottop&blast_rank=1&RID=PDGNN7ZP011) (E= 6e-153) *Alternaria alternata*  [XP_001933648.1](http://www.ncbi.nlm.nih.gov/protein/189194619?report=genbank&log$=prottop&blast_rank=3&RID=PDGNN7ZP011) (E= 2e-151) *Pyrenophora tritici-repentis* | | Brn1RTF | GCGAGTACATTCCTGGTGGT |
|  |  |  |  | Brn1RTR | GTTGACCCAGTCACCGTCTT |
| T4HN reductase (*Brn2*) | [ACIW01000092.1](http://www.ncbi.nlm.nih.gov/nucleotide/224807631?report=genbank&log$=nucltop&blast_rank=1&RID=PDES2CJ5016)  Ab022751 | [XP_001940395.1](http://www.ncbi.nlm.nih.gov/protein/189208123?report=genbank&log$=prottop&blast_rank=1&RID=PDCWCE8B01S) (E= 9e-152) *Pyrenophora tritici-repentis*  [ABK63477.1](http://www.ncbi.nlm.nih.gov/protein/118140092?report=genbank&log$=prottop&blast_rank=1&RID=PDCP50CC011) (E = 2e-149) *Cochliobolus heterostrophus* | | Brn2RTF | GGACGTAACGGAGGAGATCA |
|  |  |  |  | Brn2RTR | TCTTCGATGTGCTTGTACGC |
| Scytalone dehydratase | [ACIW01000946.1](http://www.ncbi.nlm.nih.gov/nucleotide/224806777?report=genbank&log$=nucltop&blast_rank=1&RID=PDF3T50E011)  Ab03393.1 | [ABK63478.1](http://www.ncbi.nlm.nih.gov/protein/118140094?report=genbank&log$=prottop&blast_rank=1&RID=PDFNE46J011) (E= 3e-99) *Cochliobolus heterostrophus*  [XP_001935090.1](http://www.ncbi.nlm.nih.gov/protein/189197505?report=genbank&log$=prottop&blast_rank=2&RID=PDFNE46J011)(E =5e-99), *Pyrenophora tritici-repentis* | | Scd1RTF | GAGGACGAGATTGTCGGGTA |
|  |  |  |  | Scd1RTR | ACTCGAACCACCTGATGTCC |

Table S6B. List of primers used for transformation constructs

| Primer name | Lab stock | Sequence (5’ to 3’) |
| --- | --- | --- |
| P1 | 1Amr1L5F1 | GCAGAAAGCTTCTCGTGTCC |
| P2 | HygFc-Amr1L5 | ATCAGTTAACGTCGACCTCGAGGTTCGATCAAGTGGGATG |
| P3 | 3AmrLRcHygF | CATCCCACTTGATCGAACCTCGAGGTCGACGTTAACTGAT |
| P4 | 4CmrL3FHygR | CATATCCTCGCCCGTAGTGTCGTCGACGTTAACTGGTTCC |
| P5 | 5HygRc-AmrL3F | GGAACCAGTTAACGTCGACGACACTACGGGCGAGGATATG |
| P6 | 6cmr1-L3R | CAACGTCTGCTTGGAACTGA |
| P1' | 1’Cmr1L5F1 | CTCACCGCTACCAGACACCT |
| P2' | 2’HygFc-Amr1L5F1 | CTCGCCCTTGCTCACCATTCTGGTGAACGACTGTCCA |
| P3' | 3’Amr1L-RcHygF | TGGACAGTCGTTCACCAGAATGGTGAGCAAGGGCGAG |
| P7 | TrpPF | ACCTCTGGCTGGAGGTCAC |
| P8 | Amr3FcpTrpR | AATATGTGCAGAAGACCATGTTGTTTGGATGCTTGGGTAGAATAG |
| P9 | 5ptrpRc5Amr3F | CTATTCTACCCAAGCATCCAAACAACATGGTCTTCTGCACATATT |
| P10 | 1Amr1GFP-GA | TAACATCGTGCAGCCTCATC |
| P11 | 2’Amr1GFP | CAGCTCCTCACCCTTACTCACGCCATCGGAGAATCTGGTAG |
| P12 | 6Amr1GFP-GA | CAATTACCTACGCGACACGA |
| P13 | 3’Amr1GF | CTACCAGATTCTCCGATGGCGTGAGTAAGGGTGAGGAGCTG |
| P14 | 4z9619-3FcHygR | AAACCAAAATGCCAATCTCGCGTCGACGTTAACTGGTTCC |
| P15 | pNR-20F | AAAGGGAACAAAAGCTGGAG |
| P16 | pNR-775R | CCTCGAGGTCGACGGTATC |
